# m5C regulator-mediated methylation modification phenotypes characterized by distinct tumor microenvironment immune heterogenicity in colorectal cancer

**DOI:** 10.1038/s41598-023-37300-z

**Published:** 2023-07-24

**Authors:** Zhihua Chen, Quanfa Li, Yilin Lin, Suyong Lin, Ji Gao, Shaoqin Chen

**Affiliations:** 1 Department of Gastrointestinal Surgery, The First Affiliated Hospital, Fujian Medical University, Fuzhou, 350005 China; 2 Peking University People’s Hospital, Beijing, 100044 China; 3 Fujian Provincial Key Laboratory of Precision Medicine for Cancer, The First Affiliated Hospital, Fujian Medical University, Fuzhou, 350005 China; 4 School of Basic Medicine Sciences, Fujian Medical University, Fuzhou, 350122 China

**Keywords:** Colorectal cancer, Cancer epigenetics

## Abstract

The RNA 5-methylcytosine (m5C) modification has been demonstrated to be an important epigenetic regulator and to impact colorectal cancer (CRC) progression. However, the potential roles of m5C modification in immune cell infiltration in the CRC tumor microenvironment (TME) remain unknown. The m5C modification phenotypes were comprehensively evaluated based on 14 m5C regulators in a meta-CRC cohort of 1792 patients and systematically correlated with the m5C modification phenotypes, immune cell infiltration characteristics and known biological processes. The m5Cscore model was constructed by principal component analysis (PCA) algorithms to quantify the m5C modification phenotypes of individual CRC samples and was used to predict the immunotherapy response. We identified three m5C modification phenotypes associated with distinct clinical outcomes and biological processes among the 1792 meta-CRC patients. Three phenotypes with a highly consistent TME landscape and characteristics were revealed: immune excluded, immune desert and immune inflammation. The meta-CRC patients were divided into high and low m5Cscore subgroups based on the m5Cscore. The m5Cscore was confirmed to have a negative correlation with infiltrating immune cells and PD-L1 expression and a positive correlation with tumor mutation burden (TMB), mutation rate and microsatellite instability (MSI) score. Moreover, patients in the low m5Cscore group had better immunotherapy responses and significant durable survival benefits in independent anti-PD-1/L1 immunotherapy cohorts for the immune checkpoint inhibitor (ICI) therapeutic strategy. This study revealed that m5C modification plays a crucial role in TME composition and complexity. Comprehensive evaluation of the m5C modification phenotypes of individual patients will enhance our understanding of TME characteristics and promote the application of more appropriate and personalized treatment strategies.

## Introduction

More than 170 modifications of RNA modifications have recently emerged as an important regulatory layer of gene expression, and these include the main modifications N6-methyladenosine (m6A), 5-methylcytosine (m5C) and N1-methyladenosine (m1A)^1^. RNA modifications play a crucial role in a wide range of pathophysiological processes and numerous diseases, especially the development of cancer^2,3^. Recently, with the rapid development and improvement of detection technologies, increasing attention has been given to m5C modification and its biological function and implications^4^. m5C modifications affect and regulate mRNA molecule maturation, stability and translation processes through three types of regulators, which were identified to maintain m5C modification as a dynamic and reversible process^5–7^. Regulators include the “writers” that mediate m5C modification, the “readers” that recognize m5C modification to maintain and participate in mRNA stability, translation and splicing, and the “erasers” that remove m5C modifications restore to original mRNA structures. We further explored and revealed the m5C modification mechanisms in gene regulation via in-depth investigation of the regulators. Increasing evidence has demonstrated that genetic changes and abnormal expression of m5C regulators are correlated with malignant tumor formation and progression and immunomodulatory abnormalities^8–11^. A comprehensive investigation and understanding of the expression perturbations and genetic variation of m5C regulators based on cancer heterogeneity will further aid in the identification and identification of beneficial m5C modification-based therapeutic targets.

Despite significant reductions in the incidence and mortality of colorectal cancer (CRC) over several decades, CRC^12^ is still the third most commonly diagnosed cancer and the second most common malignancy worldwide because of its high rates of tumor recurrence and metastasis, which cause a poor prognosis^13,14^. Systemic treatments, including surgery, chemotherapeutics and targeted therapeutics, have reached a bottleneck for improving patient prognosis. Immunotherapies, such as programmed death-1 (PD-1) and cytotoxic T-lymphocyte antigen 4 (CTLA-4) inhibitors, have transformed the treatment landscape for CRC patients and become important treatments, especially for advanced CRC patients with microsatellite instability-high (MSI-H) disease, which is highly infiltrated with immune cells and carries a high neoantigen load^15^. Increasing evidence has demonstrated that the diversity and complexity of tumor microenvironment (TME) components not only crucially affects tumorigenesis and progression but also affects the response to immunotherapy^16^. The TME includes cancer cells, infiltrated immune cells, stromal cells, and secreted cytokines, which are affected by complex interactions and regulatory mechanisms. The TME is classified as immune “hot”, “cold” or “excluded” to describe the major immune coordination profiles as a novel concept of “immune contexture” in CRC tumors, which can help not only represent and identify the different TME characteristics but also guide treatment options and evaluate the immunotherapy response^17–19^. It is very important to identify the TME immune phenotypes by comprehensively analyzing and clarifying the components of the TME and the interaction mechanisms in order to predict the immunotherapeutic response and guide treatment. Therefore, evaluating infiltrated immune cells and distinguishing tumor immune phenotypes based on the characteristics and internal composition of the TME is an important method and approach to develop novel potential immunotherapeutic strategies^20,21^.

Increasing studies and evidence have revealed that multiple crosstalk mechanisms of m5C modification play a role in regulating and recasting infiltrated immune cells in the TME. Li Shuangqi^22^ found that a TET2-mediated IL-6/G-MDSCs/CD8 T-cell immune response cascade safeguards host adaptive antitumor immunity. TET2-knockout mice have increased IL-6 levels in the seeded tumor tissue, which in turn stimulates the recruitment of immunosuppressive granulocyte myeloid-derived suppressor cells (G-MDSCs) and reduces the number of CD8 T cells during antitumor therapy. Thus, systemic knockout of Tet2 in mice resulted in accelerated tumor growth and was able to impede the anti-PD-1 antibody treatment response. Peng Ding reported that the loss of TET2 reduced T-cell infiltration in the TME and impaired the expression of cytokines and chemokines; he also found that TET2 was recruited to IRF1 with IFN-γ-STAT1 signaling and maintains IRF1 is to stimulate PD-L1 expression^23^. More results demonstrated the crucial role of TET2 in remodeling the TME and improving immunotherapy response. Y-box binding protein-1 (YBX1) is the most common tumor-associated antigen and induces a T-cell response^24^. In the current study, YBX1 positively improved the immune infiltrates of CD4 Th2 cells in oral squamous cell carcinoma (OSCC) to promote IL-4 expression, which reduced cell apoptosis in OSCC. YBX1 is negatively correlated with OSCC patient prognosis^25^. However, most of the current research is limited to one or two m5C regulators, but the development and remodeling of the tumor TME is regulated in a highly coordinated manner by multiple regulators and multiple molecules. In recent years, with the continuous development of sequencing technology, many useful transcriptomic and genomic data have been accumulated on many open platforms, which provide an ideal resource for the comprehensive analysis and study of m5C regulators and the immune regulation landscape^4^. Therefore, a comprehensive understanding of the TME cell infiltration properties and TME landscape that are coordinated and mediated by multiple m5C regulators will help to enhance our understanding of tumor immunotherapy.

In this study, we comprehensively assessed the intrinsic relationship between the m5C modification pattern and TME cellular immune infiltration landscapes and characteristics by integrating genome, transcriptome, somatic mutation and other multidimensional data for 1792 CRC samples from The Cancer Genome Atlas (TCGA) and Gene Expression Omnibus (GEO) databases. The CRC patients were classified by ConsensusClusterPlus clustering according to three distinct m5C modification patterns whose TME characteristics were closely associated with three previously reported immunophenotypes: immune inflammation, immune desert and immune excluded^19^. Furthermore, we constructed a scoring scheme to quantify the m5C modification patterns of individual tumors and predict the immune checkpoint inhibitor (ICI) response for CRC patients. These findings revealed that m5C modifications play an integral role in the development of distinct tumor immune microenvironment characteristics and guiding therapeutic intervention strategies for CRC.

## Materials and methods

### Collection and preprocessing of the CRC source dataset

The entire analysis workflow is shown in Fig. S1. To obtain RNA-sequencing data for colon adenocarcinoma (COAD), rectal adenocarcinoma (READ) and normal colorectal tissues, matching clinical data were downloaded from the TCGA (https://portal.gdc.cancer.gov/) and the GEO (www.ncbi.nlm.nih.gov/geo/). In total, data for 1792 CRC cases in the 5 eligible CRC cohorts (TCGA-CRC (COAD and READ), and GSE39582, GSE37892, GSE14333 and GSE161158) were gathered in this study for further analysis. Then, FPKM values were transformed into transcripts per kilobase million (TPM) values, and the GEO datasets were assessed via a robust multiarray averaging method with the simpleaffy and affy packages for background adjustment and quantile normalization. We excluded patients with missing overall survival (OS) values or short OS times (< 30 days). Finally, the baseline information of all eligible CRC patients is summarized in Table 1, and the m5C regulator function type and expression levels in the meta-CRC (GEO-CRC and TCGA-CRC datasets) dataset are listed in Tables S1 and S2. The somatic mutation data were acquired from the TCGA database. The R package “Rcircos” was employed to plot the copy number variation landscape of 14 m5C regulators in human chromosomes. The number of nonsynonymous mutation (including in-frame mutation, missense mutation, frameshift mutation, nonsense mutation, and splice site mutation) was recognized as the tumor mutation burden (TMB). All of the data were analyzed with R software (version 4.1.3).

**Table 1:** The clinical characteristics of the colorectal cancer (CRC) patients included in this study.

Characteristics (n = 1792)	GEO-CRC (n = 1255)				TCGA-CRC (n = 537)
	GSE39582 (n = 585)	GSE161158 (n = 250)	GSE14333 (n = 290)	GSE37892 (n = 130)	
Age					
≤ 65	228 (39.00%)	126 (50.40%)	133 (45.86%)	55 (42.31%)	234 (43.58%)
> 65	357 (61.00%)	124 (49.60%)	157 (54.14%)	75 (57.69%)	303 (56.42%)
Gender					
Male	322 (55.04%)	–	164 (56.55%)	69 (53.08%)	287 (53.45%)
Female	263 (44.96%)	–	126 (43.45%)	61 (46.92%)	250 (46.55%)
T Stage					
T1	16 (2.73%)	–	–	–	15 (2.94%)
T2	49 (8.38%)	–	–	–	93 (17.32%)
T3	379 (64.79%)	–	–	–	366 (68.16%)
T4	119 (20.34%)	–	–	–	63 (11.73%)
Unknown	22 (3.76%)				
N Stage					
N0	314 (53.67%)	–	–	–	316 (58.85%)
N1	106 (18.12%)	–	–	–	128 (23.84%)
N2	137 (23.42%)	–	–	–	92 (17.13%)
Unknown	28 (4.79)	–	–	–	1 (0.19%)
M Stage					
M0	499 (85.30%)	–	–	–	400 (74.49%)
M1	61 (10.43%)	–	–	–	75 (13.97%)
Unknown	25 (4.27%)	–	–	–	62 (11.55%)
Stage					
Stage I	42 (7.18%)	33 (13.20%)	44 (15.17%)	–	93 (17.32%)
Stage II	270 (46.15%)	76 (30.40%)	93 (32.07%)	73 (56.15%)	209 (38.92%)
Stage III	210 (35.90%)	82 (32.80%)	92 (31.72%)	57 (43.85%)	149 (27.75%)
Stage IV	61 (10.43%)	59 (23.60%)	61 (21.03%)	–	76 (14.15%)
Unknown	2 (0.34%)	–	–	–	10 (1.86%)

### Consensus molecular clustering for 14 m5C regulators

A total of 14 regulators were extracted from four integrated GEO datasets and one TCGA dataset to identify different m5C modification patterns mediated by the 14 m5C regulators. These 14 m5C regulators included 11 writers (NSUN2-7, DNMT1, TRDMT1, DNMT3A, DNMT3B and NOP2), 2 readers (YBX1 and ALYREF) and 1 eraser (TET2)^4^. Unsupervised clustering analysis was applied to construct consensus molecular clusters for 14 m5C modification patterns based on the expression of 14 m5C regulators. The characteristics of the m5C regulator cluster were further analyzed. Unsupervised clustering was performed with the ConsensusClusterPlus package, and 1000 repetitions of the outputs were aggregated to guarantee the stability of the classification of CRC samples^26^. The consensus clustering algorithm was used to select the optimal number of stable clusters^27^.

### Gene set variation analysis (GSVA)

Gene Ontology (GO, c5.go.v7.5.1.symbols) and Kyoto Encyclopedia of Genes and Genomes (KEGG, c2.cp.kegg.v7.5.1.symbols) analyses were performed with the “GSVA” R package to identify the significantly different biological processes enriched between the different m5C modification patterns. The Hallmark gene sets of KEGG and GO were downloaded from the Molecular Signatures Database (MSigDB) for GSVA. FDR < 0.25, adjusted *P* < 0.05 and qvalueFilter < 0.05 were set as the cutoff criteria for statistical significance. GO and KEGG annotation analyses of the m5C modification patterns were performed by using the clusterProfiler R package with a cutoff value of FDR < 0.01and the collection of differentially expressed genes (DEG) between the three different m5C modification patterns as the input.

### Identification of the distinct differentially expressed genes (DEGs) between m5C modification phenotypes

To identify m5C modification-related genes, we collected the m5C modification-related DEGs between the distinct m5C modification phenotypes that were previously clustered and classified based on the expression of the 14 m5C regulators. The limma R package was applied to identify the distinct DEGs between different m5C modification phenotypes^28^. The significance criteria for determining distinct DEGs was set as an adjusted *P* value less than 0.001.

### Quantification of the m5C modification patterns and construction of the m5Cscore model

To quantify the m5C modification patterns of each meta-CRC tumor, we used the set of scoring systems that were reported in the gastric cancer m6A gene signature previously, and we followed the methods of Bo Zhang^29^ and Wei Chong et al.^30^. We established the m5C gene signature to evaluate the m5C modification pattern of the meta-CRC, and we constructed the m5Cscore model to quantify the m5C modification patterns. First, the overlapping distinct DEGs identified from different m5C clusters were selected and identified with univariate Cox regression analysis as prognosis-related DEGs with a *P* value < 0.01. Then, the unsupervised clustering method was utilized based on the prognosis-related DEGs to classify the meta-CRC patients into different groups for further analysis. We collected the significant prognosis-related DEGs. The number of unsupervised gene clusters was defined with the consensus clustering algorithm for optimal stability. Principal component analysis (PCA) was utilized to construct the m5C gene signature, and principal components 1 and 2 were selected and extracted as the m5C gene signature score. This method not only primarily focused the signature score on the set with the largest group of well-correlated (or anticorrelated) genes in the set but also downweighted the contributions of genes that did not correlate well with other set members. Finally, the m5Cscore was calculated using a method similar to previous studies^31^: m5Cscore = ∑(PC1i + PC2i), where i is the expression of m5C phenotype-related genes.

### Correlations among m5C clusters and gene clusters and other known biological processes

There are some known biological processes that can be evaluated according to biological gene signatures^32^, which have been used and proven to be valid^33,34^. These signature are related to (1) angiogenesis; (2) antigen processing machinery; (3) CD8 + T effector; (4) cell cycle; (5) DNA damage repair (gene expression profiling, G); (6) DNA damage repair (mutational profiling, M); (7) DNA replication; (8) epithelial mesenchymal transition (EMT) signatures (EMT1, EMT2 and EMT3); (9) Fanconi anemia; (10) FGFR3-related genes; (11) homologous recombination; (12) immune checkpoint; (13) KEGG discovered histones; (14) mismatch repair; (15) nucleotide excision repair; (16) Pan-Fibroblast TGFb response signature (Pan-F-TBRS); and (17) WNT targets. All of the genes included in the signatures of the known biological processes are shown in Table S2. We studied in detail the specific association between the enrichment scores of each known biological pathway and the m5C clusters, the gene clusters and the m5Cscore, collectively.

We also collected the gene signatures of the growth factor TGF-β/EMT pathway (including TWIST1, TGRB1, CLDN3, SMAD9, ACTA2, TGFBR2, COL4A1, VIM and ZEB1-AS1), immune activation-related genes (including CXCL10, TNF, TBX2, CD8A, GZMB, PRF1, CXCL9, GZMA and IFNG) and 44 immune checkpoint genes (including PD-1, PD-L1, PD-L2, CD80, CTLA-4, CD40, CD86, CD70, IDO1, HHLA2, LAG3, HAVCR2 and TIGIT) from the published literature^31,35^. The growth factor TGF-β/EMT pathway and immune activation-related gene signature were deemed the cytokine and chemokine signatures, respectively. We extracted data for the transcripts of TGF-β/EMT pathway-related genes (Table S3), immune activation-related genes (Table S4) and immune checkpoint genes (Table S5) for the meta-CRC patients. The data for transcripts of the gene signatures were extracted, and the relationships among the gene signatures, the m5C clusters, the gene clusters and the m5Cscore were investigated using the "ggpubr" package.

### Visualization of the mutational landscape of m5C regulators and modification-related genes

The TMB and mutational signatures of the individual TCGA-CRC patients were extracted from the TCGA genomic data and uploaded into the “maftools” package. The mutational landscape of m5C regulators and m5C phenotype-related genes was visualized and depicted by the waterfall function of the R “maftools” package in the TCGA-CRC cohort. The correlation between TMB and the m5Cscore was depicted by using the “ggpubr”, “reshape2” and “vioplot” R packages.

### Analyses of the TME and ESTIMATE score

Differential cluster analyses of the immune score, ESTIMATE score, stromal score, and tumor purity score were performed in the different m5C modification patterns and the m5Cscore groups based on the results of the ESTIMATE algorithm (using the “limma” and “vioplot” R packages). The single-sample gene-set enrichment analysis (ssGSEA) algorithm was used to quantify the relative abundance of 23 immune cell types in the CRC TME; these cell types included activated CD8 T cells, activated CD4 T cells, macrophages, regulatory T cells, activated dendritic cells, and natural killer T cells. The deconvolution scores were calculated with CIBERSORT 44 (http://cibersort.stanford.edu/) in ssGSEA, and the differences in each TME-infiltrating cell in the meta-CRC cohort were compared. We also determined the immune infiltration statuses of each TCGA-CRC patient via analyses with CIBERSORT, CIBERSORT-ABS, TIMER, XCELL, EPIC, and MCPCOUNTER in TIMER2.0 (http://timer.cistrome.org/).

### Verification of the immune response predictors: MSI, checkpoint gene expression, immunophenoscore (IPS) and TIDE score

The MSI data of the individual TCGA-CRC patients were extracted from the TCGA genomic data, and the relationship between MSI and the m5Cscore was depicted by using the “ggpubr”, “reshape2” and “vioplot” R packages. Checkpoint gene expression was extracted for each m5Cscore group, and the expression differences were detected with the “limma” and “vioplot” R packages. The IPS is known as a superior predictor of the response to ICIs, such as anti-PD-1 and anti-CTLA-4 indicators^36^. IPSs were also used to characterize the intratumoral immune landscapes and cancer antigenomes and quantify the determinants of tumor immunogenicity. The scoring scheme, which is a machine learning-based algorithm, was calculated based on the samplewise Z scores of four types of immune-related genes, including checkpoints or immunomodulators (CPs), MHC-related molecules (MHCs), suppressor cells (SCs) and effector cells (ECs). The IPS data of the TCGA-CRC cohort were extracted from The Cancer Immunome Atlas (TCIA), and the relevant datasets included “ips_ctla4_neg_pd1_neg”, “ips_ctla4_pos_pd1_neg”, “ips_ctla4_neg_pd1_pos” and “ips_ctla4_pos_pd1_pos”. The Tumor Immune Dysfunction and Exclusion (TIDE) algorithm considers the exclusion and dysfunction of tumor infiltration cytotoxic T lymphocytes (CTLs) by immunosuppressive factors and was utilized to predict the response to ICIs of meta-CRC patients^37^. A higher TIDE score indicated that tumor cells were more likely to induce immune escape, thus indicating a lower response rate to ICI treatment.

### Verification of the ability of the m5Cscore to predict the efficacy of ICI therapy

To verify the ability of the m5Cscore to predict the efficacy of ICI therapy in the ICI-based cohorts, we systematically searched and finally selected two ICI cohorts with complete gene expression profiles and clinical information for further study. The IMvigor210 cohort (348 patients with advanced urothelial cancer) was treated with the anti-PD-L1 antibody atezolizumab^32^, and the GSE78220 cohort (27 patients with metastatic melanoma) was treated with the anti-PD-1 antibody pembrolizumab^38^. The complete expression data and detailed clinical annotations of the IMvigor210 cohort can be obtained from http://research-pub.Gene.com/imvigor210corebiologies. The DESeq2 R package was used to normalize the raw count data of the IMvigor210 cohort, which were transformed into TPM values for further statistical analysis. The limma package was used to standardize the raw count data of the GSE78220 cohort, and the FPKM data of the gene expression profiles were converted to TPM values for greater comparability between the samples.

### Statistical analysis

All statistical analyses were performed using R software (version 4.1.2). X^2^ or Fisher’s exact test was used to compare the categorical variables between groups. The waterfall function of the maftools package was adopted to visualize the mutation landscape of the m5C cluster and m5Cscore groups in the TCGA-CRC cohort. The copy number variation (CNV) landscape of the 14 m5C regulators in 23 pairs of chromosomes was assessed by the R package RCircos. The median overall survival (OS) was analyzed by the Kaplan–Meier (K-M) method, and the cutoff point of each m5C cluster or gene cluster group was determined using the survminer R package. The significance of survival differences was determined by the log-rank test. Correlation coefficients between the expression of m5C regulators, MSI, TMB and the m5Cscore and the expression of m5C regulators and the levels of TME-infiltrating immune cells were computed by distance correlation analyses and Spearman’s test. Kruskal–Wallis tests and one-way ANOVA were used to conduct difference comparisons of three or more groups. To obtain the maximum rank statistic, the “surv-cutpoint” function of the m5Cscore was repeatedly tested for all of the potential cutoff points, and the best cutoff was used to divide the meta-CRC patients into low and high m5Cscore groups. The univariate Cox regression model was adopted to calculate the hazard ratios (HRs) for m5C regulators and m5C phenotype-related DEGs. The forestplot R package was adopted to visualize the results of univariate and multivariate Cox correlation analysis and multivariate prognostic analysis of the m5Cscore in the meta-CRC cohort.

## Results

### Visualization of the genetic variation in m5C regulators in CRC

**Figure 1:**
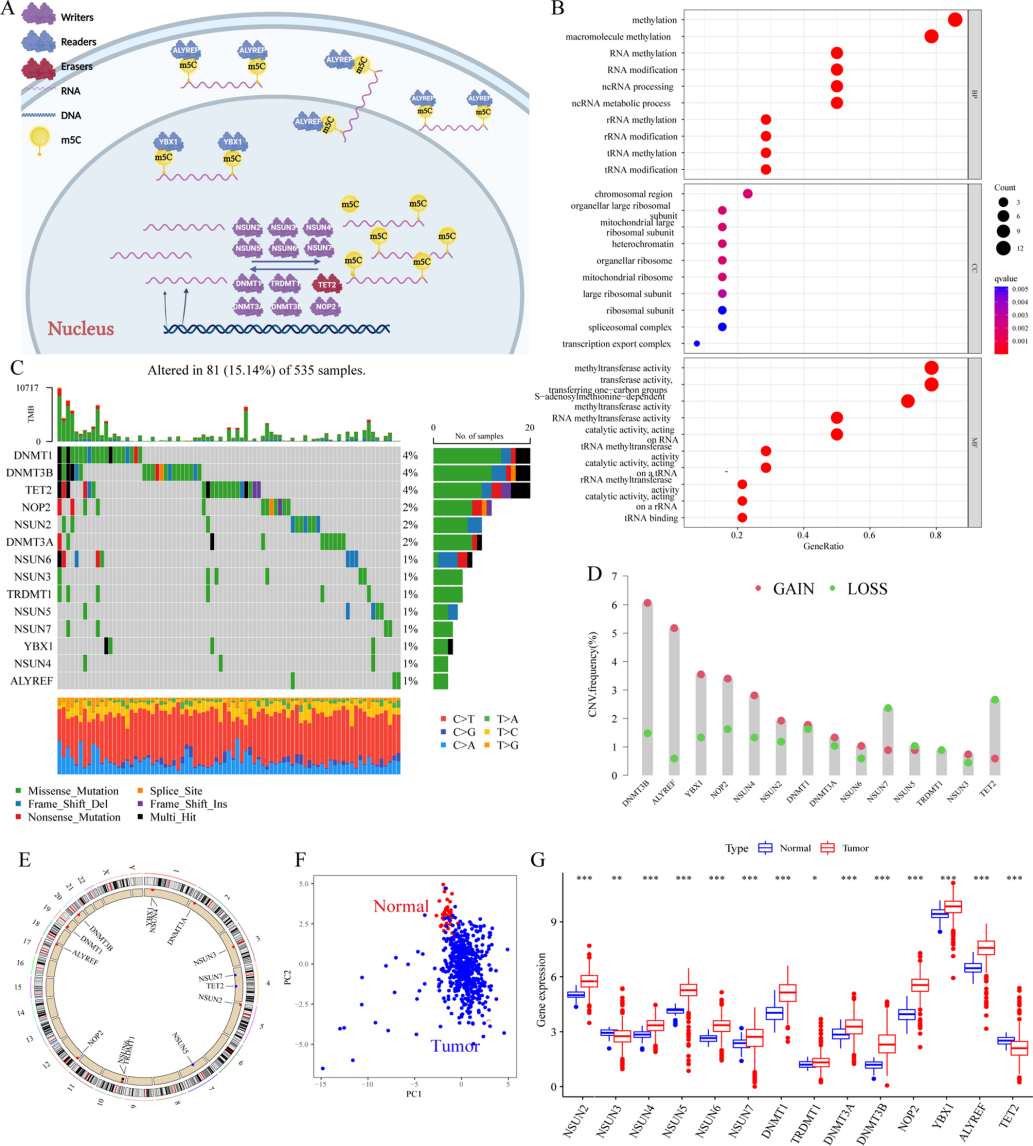
The landscape of the genetic alterations and expression of the 14 m5C regulators in CRC. (**A**) Summary of the regulation of m5C modification and its potential biological functions in RNA metabolism by m5C regulators (writer, reader and eraser proteins). (**B**) GO biological process enrichment analysis results of 14 m5C regulators. (**C**) A 15.14% mutation frequency of 14 m5C regulators was observed in 535 patients with CRC from the TCGA-CRC cohort. The individual patients are represented in each column. The upper bar plot is TMB. The mutation frequency for each regulator is shown on the right. The proportion of each variant type is shown on the right bar plot. The fraction of conversions in each patient is shown on the stacked bar plot below. (**D**) The CNV mutation variation of 14 m5C regulators is presented in the TCGA-CRC cohort. The column is the CNV alteration frequency. The green dot is the deletion frequency; the pink dot is the amplification frequency. (**E**) The location of CNV alterations in m5C regulators on 23 chromosomes in the TCGA-CRC cohort. (**F**) PCA of the expression of m5C regulators was used to distinguish tumor samples from normal samples for the TCGA-CRC cohort. Tumor: blue dot; normal: red dot. (**G**) The differential expression of 14 m5C regulators between normal and tumor tissues of the 1609 meta-CRC patients. Normal: blue dot; tumor: red dot. (*P < 0.05; **P < 0.01; ***P < 0.001).

At the beginning of this study, we researched the roles of 14 m5C regulators, including 11 writers (NSUN2-7, DNMT1, TRDMT1, DNMT3A, DNMT3B and NOP2), 2 readers (YBX1 and ALYREF) and 1 eraser (TET2), in CRC. The biological processes of the m5C regulators, such as RNA translation, mRNA stability, RNA splicing and RNA degradation, and the dynamic reversible processes of the m5C regulators that can add, remove and add m5C-modified sites are summarized in Fig. 1A. Figure 1B shows the GO biological process enrichment analysis results of 14 m5C regulators which were most enriched in the “methylation”, “RNA methylation”, and “RNA modification” terms. We first summarized the prevalence of somatic mutations and copy number variations of 14 m5C regulators in CRC. The frequency of somatic mutations of the m5C regulators was 15.14% (81/535). DMNT1, DNMT3B and TET2 showed the highest mutation frequency of 4% (Fig. 1C) in CRC samples. The mutation co-occurrence relationships across all m5C regulators are shown in Fig. S2A, and significant co-occurrence relationships were identified between NSUN4 and TET2, TRDMT1 and NSUN3, and so on. Figure 1A shows the further investigation of CNV mutations that were prevalent in the 14 m5C regulators, and most were CNV amplifications affecting factors such as DNMT3B, ALYREF, YBX1 and NOP2. In contrast, prevalent CNV deletions were most often found in TET2 and NSUN7. The locations of CNV alterations of 14 m5C regulators on chromosomes are shown in Fig. 1E. Moreover, we found that the CRC samples were completely distinguished from normal samples according to principal component analysis (PCA) based on the expression of the 14 m5C regulators (Fig. 1F). Further investigation demonstrated that the expression of all the regulators in the CRC samples was different from that in normal samples; only the expression of TET2 and NSUN3 was significantly downregulated, while the expression of the others was significantly higher in CRC samples versus normal samples (Fig. 1G). By comprehensive analysis of the inherent relationship between m5C regulator expression and CNV alteration, we found that the expression of most m5C regulators was significantly increased in CRC samples because most m5C regulator CNVs were amplification, while TET2 was significantly downregulated in CRC samples (Fig. 1D,G). We also comprehensively analyzed the inherent relationship between m5C regulator expression and somatic mutations, and the relationships between m5C regulator expression levels and somatic mutations are highlighted within the results for all the mutations reported in Fig. 1C. ALYREF and NSUN4 were significantly upregulated, while NSUN6 and DNMT3B were significantly downregulated in the mutation group versus the wild-type group (Fig. S2B–O). The above analyses demonstrated the high heterogeneity and connections of the genetic and transcriptomic landscape in m5C regulators between CRC and normal samples. Based on these results, we hypothesized that the expression alterations in m5C regulators may play an important role in CRC occurrence and progression.

### Identification of m5C regulator methylation modification patterns

**Figure 2:**
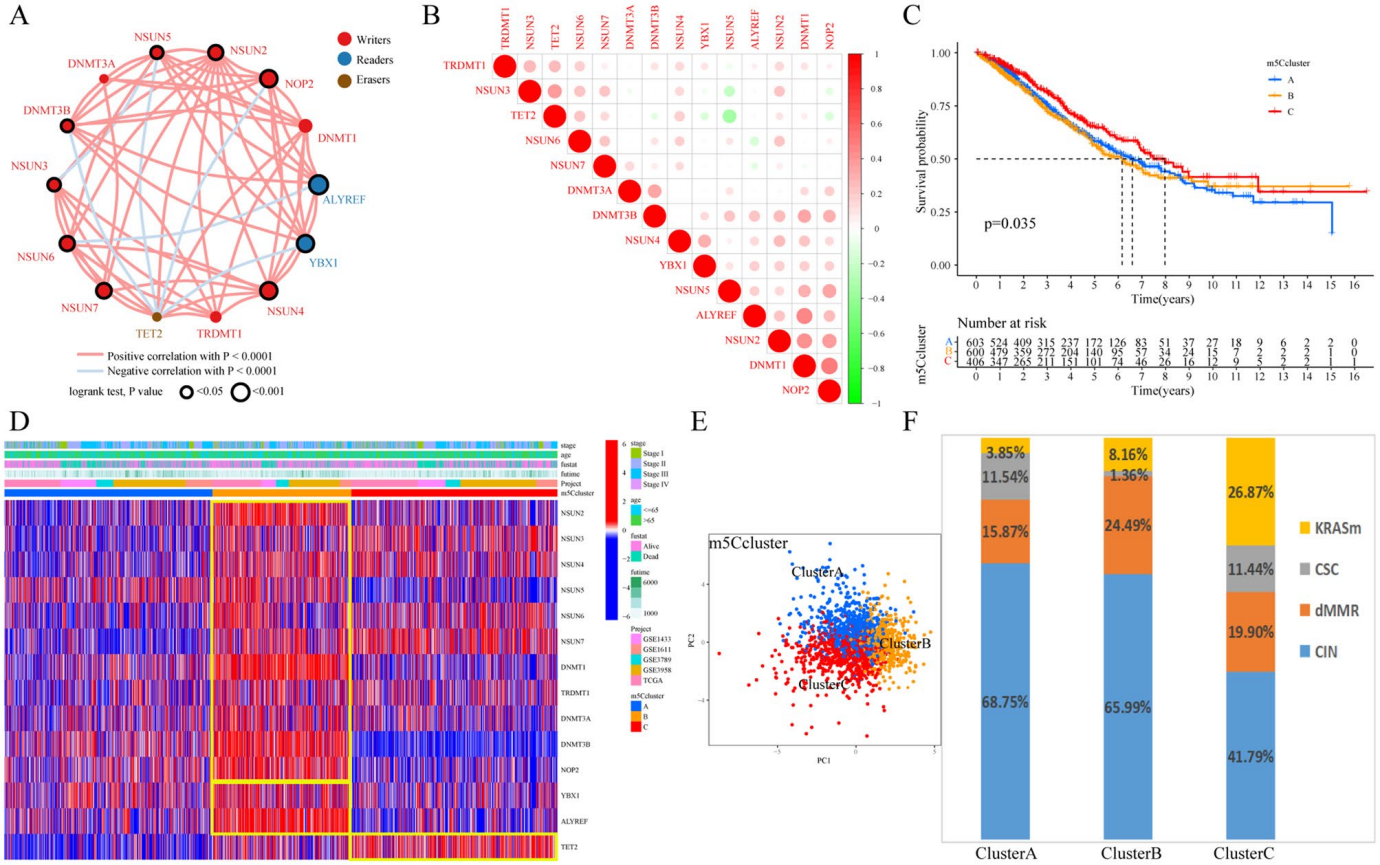
The distinct patterns of m5C methylation modification and relevant biological characteristics. (**A**) The interaction of 14 m5C regulator expression in CRC. The circle size represents the effect of the m5C regulator on CRC prognosis calculated by the KM truncation value method (respective from *P* < 0.001 and *P* < 0.05). Risk prognostic factors: purple dots in the circle; protective prognostic factors: green dots in the circle. The interactions of regulators are shown as linking lines, and the thickness shows the correlation strength of the regulators. Positive correlations are marked with red, and negative correlations are marked with blue. The red brown and yellow are erasers, readers and writers, respectively. (**B**) The correlations between these m5C regulators were calculated in CRC using Spearman correlation analysis. The negative correlation is marked with green, and the positive correlation is marked with red. (**C**) The OS for different m5C clusters of 1609 CRC patients in the meta-CRC cohort with the K-M method (log-rank test). The numbers of patients with the m5C cluster A, m5C cluster B and m5C cluster C phenotypes were 603, 409 and 600, respectively. (**D**) Unsupervised clustering of 14 m5C regulators in the five independent CRC cohorts. The clinical characteristics and CRC cohort were distributed in distinct m5C clusters. Each column represents a patient, and each row represents an m5C regulator. (**E**) PCA for the three m5C regulator modification patterns. The three m5C clusters of the meta-CRC cohort were well distinguished based on the transcriptome of m5C regulators. (**F**) The distribution of molecular subtypes of CRC in the three m5C modification patterns in the GSE39582 cohort.

A total of 1609 CRC patients with clinical annotations and available survival data were enrolled in the meta-CRC cohort (including TCGA-CRC and GEO-CRC). The mutual regulation relationships among the 14 m5C regulators were evaluated with Spearman correlation analysis (Fig. 2B). The expression of most of the m5C regulators showed a positive correlation with each other, but the eraser TET2 showed a significant negative correlation with other m5C regulators include NSUN5, DNMT3B, NOP2 and YBX1 (Fig. 2A). The relationship between the 14 m5C regulators and the prognosis of meta-CRC patients was inspected by univariate Cox regression analysis, and the results are shown in the forest plot (Fig. S3A); only ALYREF had a significant *P* value in the univariate cox regression analysis. The reader ALYREF and other regulators, such as YBX1, DNMT1, DNMT3B, NOP2, NSUN2 and NSUN7, were shown to be protective factors significantly associated with better OS in CRC patients, while other regulators, such as NSUN3-6, were shown to be risk factors for significantly poorer OS with the best expression cutoff point in the K-M method. In conclusion, these molecules may have prognostic value in CRC patients. In contrast, regulators such as NSUN3, NSUN4, NSUN5 and NSUN6 were shown to be risk factors for significantly poorer OS by the K-M method (Fig. S3B–L). Furthermore, the comprehensive landscape, the interaction network and the prognostic value of the 14 m5C regulators in meta-CRC patients were depicted in the m5C regulator network (Fig. 2A). The results indicated not only that the m5C regulators with the same functions show a significant correlation in expression (Fig. 2A,B), but also that extensive interconnectedness was present among the prognostic value in the CRC patients. Overall, the crosstalk among the m5C regulators with different functions may play important roles in the formation of different m5C modification patterns and in the occurrence and development of CRC. Based on these theoretical hypotheses and speculations, unsupervised clustering analysis was applied to construct consensus molecular clusters for qualitatively different m5C modification patterns based on the expression of 14 m5C regulators. Finally, the meta-CRC patients were clustered into three distinct m5C modification pattern clusters (m5C clusters A-C), including 603 cases in m5C cluster A, 600 cases in m5C cluster B and 406 cases in m5C cluster C (Fig. 2C, Fig. S4A–H). We found a significant difference in OS among the distinct m5C clusters of 1609 CRC patients in the meta-CRC cohort; m5C cluster C had the best prognosis, and m5C cluster B had the poorest prognosis (*P* = 0.035, log-rank test, Fig. 2C). The differential expression of m5C regulators between distinct groups classified by m5C clusters, clinical characteristics and CRC cohort is illustrated in the heatmap (Fig. 2D, Table S6). Most writers, such as NSUN2, NSUN5, NSUN6, DNMT1, DNMT3A, DNMT3B and NOP2, as well as the readers YBX1 and ALYREF, were significantly increased in the m5C cluster B group. The writers NSUN3, NSUN4, and NSUN7 and the eraser TET2 were markedly elevated in the m5C cluster C group (Fig. S3M).

### Visualization of the TME landscapes and characterization of the three m5C modification patterns

**Figure 3:**
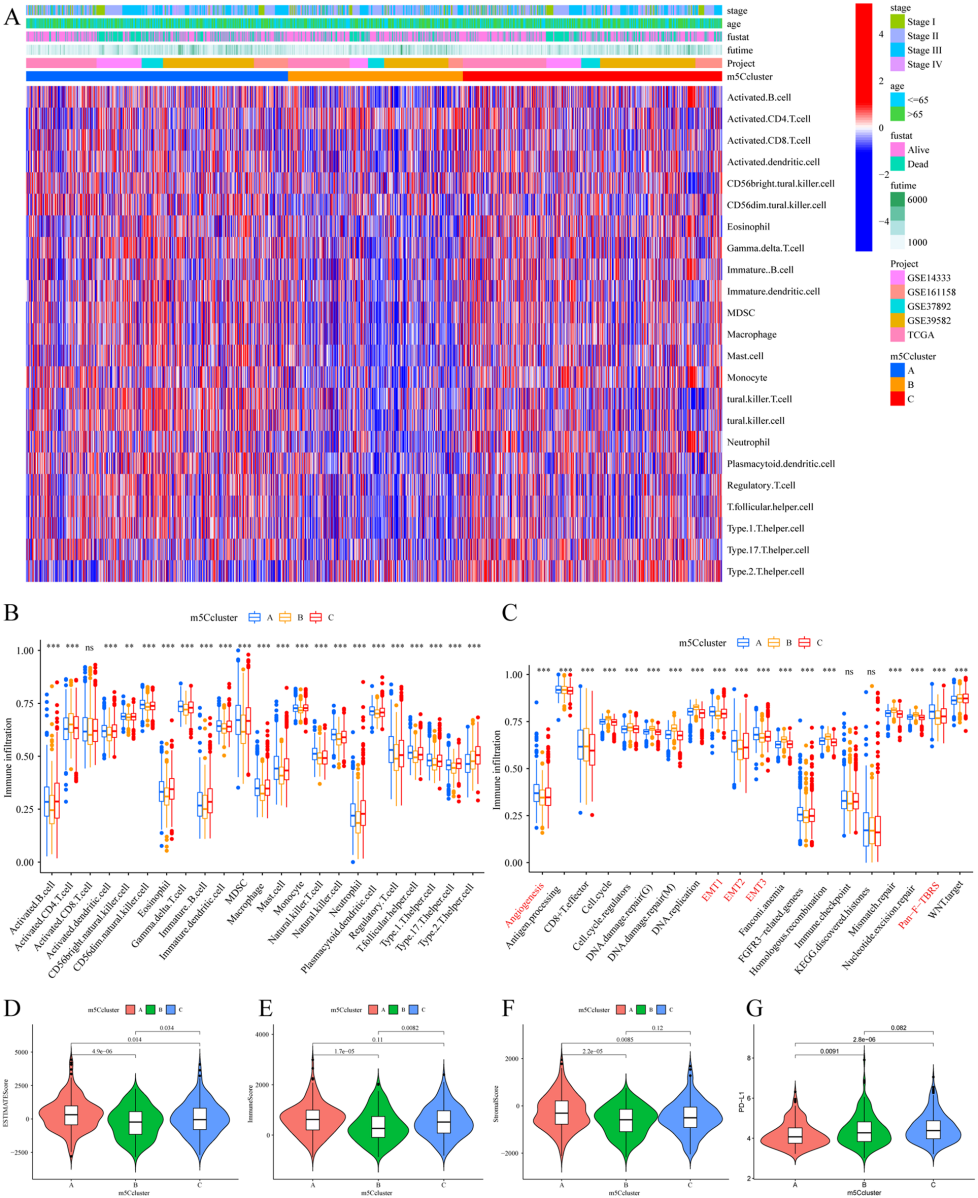
The distinct TME characteristics between the three m5C modification phenotypes. (**A**) Heatmap of the different immune cells that infiltrated the TME between the phenotypes. The clinical characteristics and CRC cohort were distributed in distinct m5C clusters. Each column represents patients, and each row represents individual immune cells. (**B**) The different immune cells infiltrated in the TME of the three m5C clusters calculated by the CIBERSORT algorithm. The Kruskal–Wallis H test was used to statistically analyze the difference between distinct m5C clusters. (**C**) The difference in the enrichment score of each known biological process gene signature between the three m5C clusters. (**D**) The immune score, (**E**) stromal score, and (**F**) ESTIMATE score of the three gene clusters were analyzed and plotted. (**G**) The checkpoint gene PD-L1 expression in the distinct m5C clusters.

The PCA landscape distinguished the CRC samples with different m5C regulator modification patterns based on the m5C clustering results (Fig. 2E). To explore the biological behaviors and functional annotations among these distinct m5C modification patterns, KEGG enrichment analysis was performed (Fig. S5A–C). The KEGG results indicated that m5C cluster A presented marked enrichment in immune activation and inflammatory processes such as the “calcium signaling pathway” and “complement and coagulation cascades”. m5C cluster B was prominently enriched in carcinogenic activation pathways, including “homologous recombination”, “cell cycle”, DNA replication” and “mismatch repair”. However, m5C cluster A showed enrichment in pathways markedly associated with stromal metabolism. Intriguingly, from the heatmap of infiltrated immune cell enrichment in the distinct m5C clusters, we found that m5C clusters A and C showed obvious infiltration of a large number of immune cells, while m5C cluster B had very little immune cell infiltration (Fig. 3A, Table S7). The infiltrated immune cells in m5C cluster A were mostly innate immune cells, such as activated B cells, CD4 + T cells, MDSCs, macrophages and mast cells (Fig. 3A, B), as well as type 1 helper T cells. m5C cluster C was extraordinarily enriched not only in innate immune cells but also in type 2 and type 17 helper T cells. However, compared to m5C cluster C patients, the m5C cluster A patients did not show survival benefits from the large number of infiltrated immune cells (Fig. 2C). Previous studies suggested that tumors with an immune-excluded phenotype always present an abundance of immune cells that are retained in the stroma surrounding tumor cell nests and scarcely penetrate their parenchyma and then play an antitumor role ^19^. Therefore, we speculated that the m5C cluster A group was the tumors of the immune-excluded phenotype because most stromal elements play a suppressive and antitumor immune effect in TEM. We also found that most of the enrichment scores of the known biological process gene signatures (Table S13) were different between the three m5C clusters, and “angiogenesis” and “EMT1-3″ had the highest scores in m5C cluster A (Fig. 3C). The distinct chemokine and cytokine characteristics of the m5C clusters were studied, and most of the immune activation-related genes and TGF-β/EMT pathway-related genes were differentially expressed among the three m5C clusters (Fig. S4I and J). TGF-β/EMT pathway-related genes were most highly expressed in m5C cluster A, while immune activation-related gene expression was not consistent. The results demonstrated that m5C cluster A showed more stromal cell activation. To further confirm our hypothesis, the ESTIMATE algorithm was used to quantify the total immune cell infiltration level, the overall stromal cell level and the tumor purity of the distinct m5C regulator modification phenotype (Fig. 3D). As expected, the evaluation score for infiltrated immune cells (immune score) in m5C cluster B was the lowest, and the evaluation score for stromal elements (stromal score) in m5C cluster A was the highest (Fig. 3E,F). The tumor purity of m5C cluster B was the highest (Fig. S5D). A previous study (in the GSE39582 cohort) classified CRC into four subtypes according to dominant molecular and respective biological characteristics: “CIN”, “dMMR”, “KRASm” (for *KRAS*-mutant) and “CSC” (for cancer stem cells). The upregulated immune system and cell growth pathways were enriched in “dMMR” tumors, while the downregulation of cell growth and death pathways and the upregulation of EMT pathways were enriched in “CSC” tumors, and the ECM-receptor interaction, Wnt and metabolic pathways and focal adhesion were enriched in “CIN” tumors^38^. The results of the m5C regulator modification phenotype analysis are consistent with the dominant molecular subtypes of the previous study; “CIN” subtype patients were mainly clustered into the m5C cluster A and B groups, whereas the “dMMR” subtype was primarily distributed in the m5C cluster B group, and the “KRASm” subtype was predominantly concentrated in the m5C cluster C group (Fig. 2F). Most of the immune checkpoint genes (30/44) were differentially expressed among the three m5C clusters (Fig. S5H). The differential expression of immune checkpoint genes such as PD-L1/PD-1 and CTLA-4 was indicated in distinct m5C clusters, which are considered biomarkers for predicting the response to anti-PD-1/L1 and anti-CTLA-4 treatment^31,37^. PD-L1 had higher expression in m5C cluster C (Fig. 3G), and CTLA-4 had higher expression in m5C cluster B (Fig. S5F). We also investigated the difference in the expression of the most other known important checkpoint genes between distinct m5C clusters, and the results revealed large differences among the three m5C clusters (Fig. S5G). Based on the results, the TME landscapes and characteristics of the three m5C modification patterns were well elucidated, and the m5C modification patterns were found to clearly reflect the different infiltrated immune cell and TME landscapes. Specifically, m5C cluster C was recognized as an immune-inflamed phenotype significantly related to infiltrating abundant immune cells and immune activation; m5C cluster B was recognized as an immune-desert phenotype significantly related to infiltrating a lack of immune cells; and m5C cluster A was recognized as an immune-excluded phenotype significantly related to activated stromal cells and decreased immune cell infiltration.

The specific correlation between each different immune cell infiltrate in the TME and each m5C regulator was further investigated by applying Spearman's correlation analyses (Fig. S8A). The expression of TET2 exhibited a significant positive correlation with most infiltrated immune cells, whereas the other m5C regulators showed a significantly negative correlation with most infiltrated immune cells. We also investigated the specific correlations between the enrichment scores of each known biological process gene signature and each m5C regulator using Spearman analyses (Fig. S8B), and the correlations were relatively consistent; the expression of most m5C regulators exhibited a significantly negative correlation with signatures including angiogenesis, EMT1, EMT2, EMT3 and Pan-F-TBRS and a positive correlation with the other biological process signatures. This may highlight the functional characteristics of the m5C regulators and the underlying mechanisms of their function.

Peng Ding found that the loss of TET2 reduced the infiltration of T cells and suppressed cytokine and chemokine expression, consequently stimulating TET2 activity, and this effect can be exploited to improve immunotherapy in renal cell carcinoma. TET2 may be recruited to IRF1 and maintain IRF1 demethylation, ultimately inducing PD-L1 expression^23^. TET2, as the only m5C eraser, attracted our attention because it exhibited a significant positive correlation with most infiltrated immune cells and a protective effect on prognosis. The role of TET2 in CRC was revealed through additional comprehensive studies. The expression of TET2 was significantly reduced in CRC tumors compared with normal tissues (Fig. 1G). Moreover, except for NSUN5, NOP2 and YBX1, most other m5C regulators were significantly positively correlated with TET2 (Fig. 2A and Fig. S9G). Most of the different immune cells that infiltrated the CRC TME were remarkably enhanced in the TET2 high expression group compared with the low expression group, and these groups were classified based on the median expression level (Fig. S9A). The ESTIMATE algorithm showed a remarkably higher stromal score and immune score in the TET2 high expression group but a significantly lower tumor purity score, which further confirmed the above finding (Fig. S9C–F). The TET2 were associated with the TGF-β/EMT pathway and immune activation-related gene signature (Fig. S9H,I). As expected, the expression of most chemokine and cytokine characterization genes was markedly increased in the TET2 high group compared with the low group, which further confirmed the important function in the infiltrating immune cells. The enrichment scores of the known biological processes, such as CD8 + T effectors, FGFR3-related genes and WNT targets, were different between the distinct TET2 expression groups (Fig. S9B), which suggested the potential pathway of action of TET2 in CRC. In addition, we found that the expression of each immune checkpoint gene was significantly higher in the TET2 high group than in the TET2 low group (Fig. S9J). In summary, TET2 may play an important role in CRC by recruiting infiltrating immune cells and stimulating cytokine and chemokine expression, and TET2 activity may be used to induce the intratumoral antitumor immune response and improve immunotherapy in CRC. These results should be confirmed by further experimental studies.

### Identification of the distinct DEGs between m5C modification phenotypes

**Figure 4:**
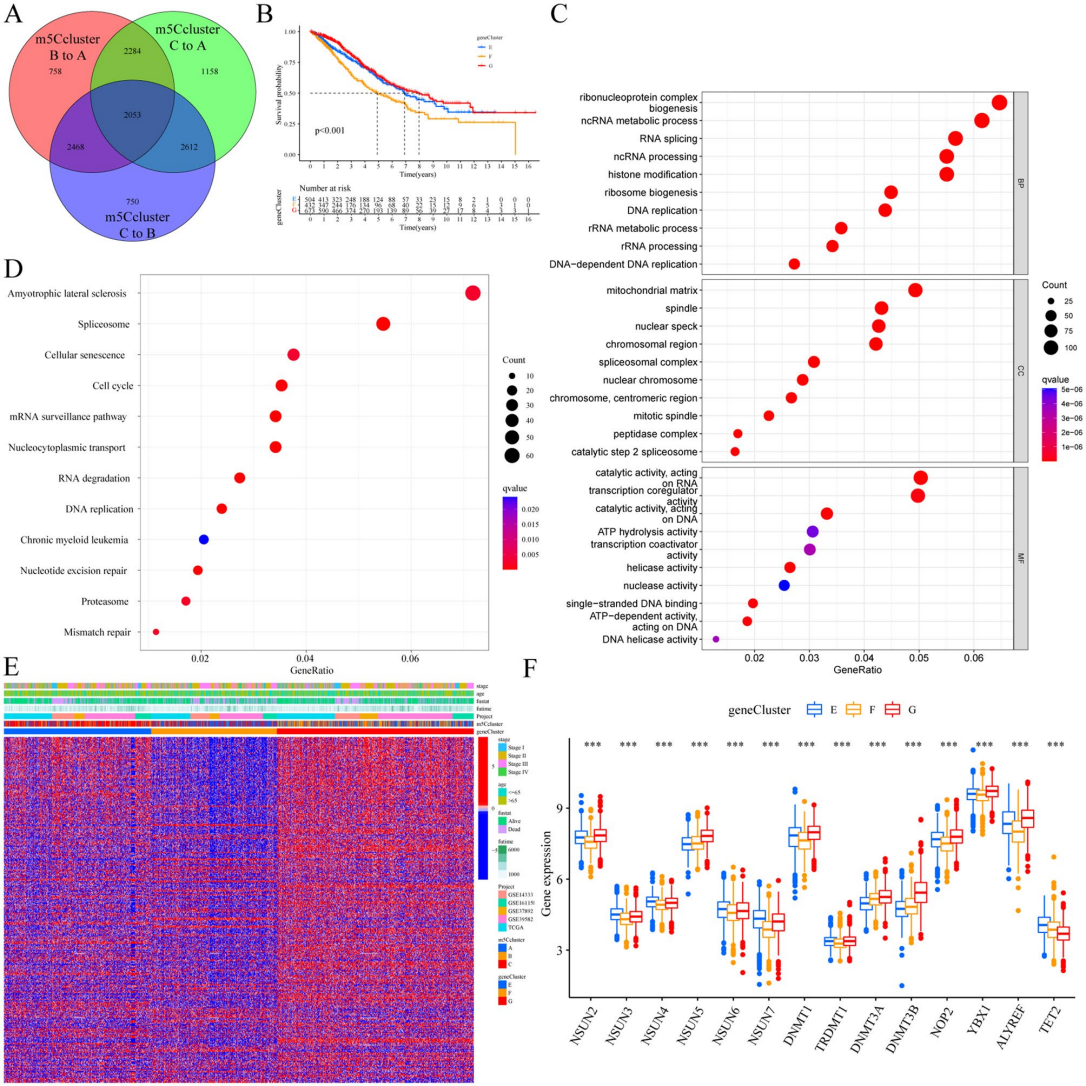
Construction of an m5C modification phenotype-related differentially expressed gene (DEG) signature and functional annotation in the meta-CRC cohort. (**A**) A total of 2053 DEGs between the three m5C clusters were identified and are shown in the Venn diagram. (**B**) The survival curves of the distinct m5C gene signature groups were analyzed by the K-M method (log-rank test). The numbers of patients with the gene cluster E, gene cluster F and gene cluster G phenotypes were 504, 432 and 673, respectively. (**C**) GO enrichment analysis and (**D**) KEGG enrichment analysis were performed; functional annotation for m5C modification phenotype-related DEGs is shown (www.kegg.jp/kegg/kegg1.html). The size of the circles represents the enrichment of the genes, and the color depth represents the q-value. (**E**) Unsupervised clustering of the m5C-related genes into clusters in the five independent CRC cohorts was performed. The clinical characteristics of CRC cohort patients in distinct gene clusters are shown. Each column represents a patient, and each row represent a DEG. (**F**) Differential expression of 14 m5C regulators between distinct gene cluster groups (*P < 0.05; **P < 0.01; ***P < 0.001).

As the underlying genetic alterations and expression interactions in the three different m5C modification phenotypes are not clear, we hoped to further study and explore the changes in the underlying m5C-related transcriptional expression information in the three m5C modification phenotypes in CRC. The limma package was applied to determine the m5C modification phenotype-related differentially expressed genes (DEGs). A total of 2053 overlapping DEGs between the distinct m5C modification phenotypes were identified as the critical distinguishing index of the distinct m5C modification phenotypes (Fig. 4A, Table S8). Univariate Cox regression analyses were applied to determine the prognostic gene signature, and 381 prognostic-related DEGs were incorporated into the m5C-related gene signature (Table S9). GO enrichment analysis of the m5C-related gene signature showed that the significantly enriched biological processes were related to RNA/DNA and histone modification, transcription, and energy metabolic activity (Fig. 4C, Table S11), and similar results were found in KEGG enrichment analysis (Fig. 4D, Table S10). Unsupervised consensus clustering analysis was performed based on the 381 prognostic-related DEGs, and three stable and specific gene phenotypes were obtained (Fig. S6A–H). Finally, the meta-CRC cases were clustered into three distinct m5C gene signature clusters (m5C gene clusters E, F and G, including 504 cases in gene cluster E, 432 cases in gene cluster F and 673 cases in gene cluster G).The critical m5C-related gene signatures of the three distinct gene clusters are illustrated in a heatmap diagram (Fig. 4E). We found significant differences in OS between the three distinct gene clusters of meta-CRC cohort patients: gene cluster G had the best prognosis and gene cluster F had the poorest prognosis (*P* < 0.001, log-rank test, Fig. 4B). The high enrichment scores of known biological processes in the gene cluster F group, such as angiogenesis, EMT1-3 and Pan-F-TBRS signatures, may suggest the potential function and reasons that this group has the poorest prognosis (Fig. S7B). As expected, the expression levels of the 14 m5C regulators were significantly different between the three gene clusters (Fig. 4F), which further confirmed the m5C regulator modification phenotype.

Further investigation was performed to reveal the role of m5C gene clusters in TME immune regulation. First, we found that all of the chemokine and cytokine characteristics in the TGF-β/EMT pathway and immune activation-related gene signature were remarkably different among the three gene clusters (Fig. S6I,J), and the expression of most genes was highest in gene cluster F, which further confirmed the abundant infiltration of immune cells. Intriguingly, the infiltrated immune cell enrichment in the distinct three gene clusters was different from that in the m5C clusters shown on the heatmap (Fig. S7C). We found that gene cluster F has the highest number of infiltrated immune cells, while gene cluster G had very little immune cell infiltration, and gene cluster E exhibited many Type 2 and Type 17 helper T cells. Moreover, the ESTIMATE algorithm was used to quantify the total TME landscape of the distinct gene clusters. As expected, the immune score and the stromal score in gene cluster F were the highest, and those in gene cluster G were the lowest (Fig. S7D,F); simultaneously, the tumor purity showed the opposite pattern (Fig. S7G). Most of the immune checkpoint genes (40/44) were differentially expressed among the three gene clusters (Fig. S7L). The expression of the checkpoint genes PD-L1, PD-1, CTLA-4 and CD80 was higher in gene cluster F (Fig. S7H–K). It is clear that the TME landscapes and characteristics are more consistent among the three gene cluster phenotypes than among the m5C modification phenotypes, indicating that the gene clusters can better display the internal differences among samples with a given m5C modification phenotype and better evaluate the differences in the TME and immunotherapy response.

### Construction of the m5Cscore model and assessment of its clinical value

**Figure 5:**
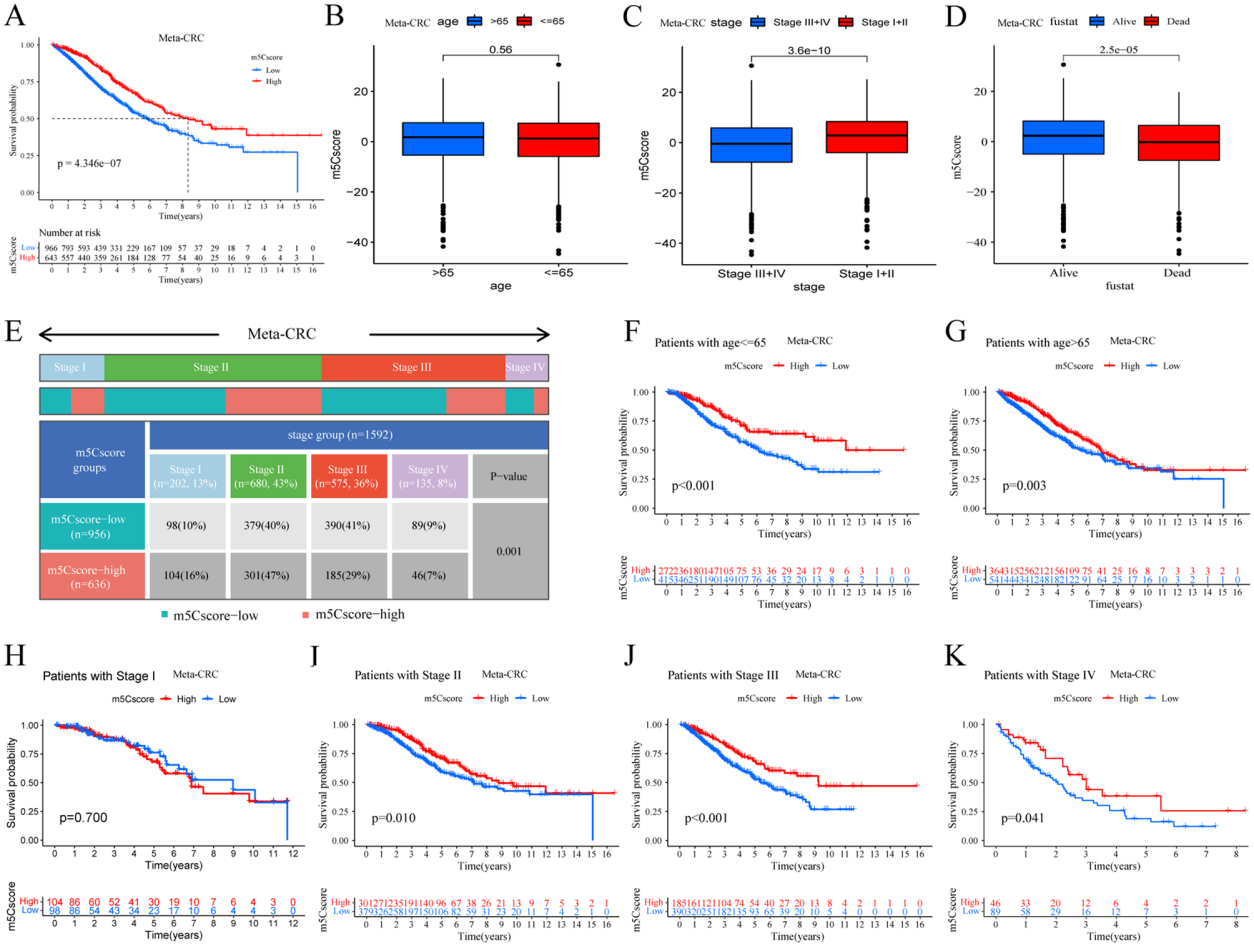
The value of the m5Cscore and clinical function in the meta-CRC cohort. (**A**) Survival time and survival status comparisons between the high- and low-m5Cscore groups (K-M method, *P* < 0.001, log-rank test). (**B**) The difference in the m5Cscore in the distinct CRC age groups (≤ 65 vs. > 65). (**C**) The difference in the m5Cscore in the distinct CRC stage groups (I + II vs. III + IV). (**D**) The difference in the m5Cscore in the distinct CRC outcome groups (alive vs. dead). (**E**) The distribution diagram of the m5Cscore in the distinct CRC stage groups. (**F**) The OS between the high- and low-m5Cscore groups in the age ≤ 65 group (687 samples) and (**G**) the age > 65 group (905 samples). (**H**–**K**) The OS between the high- and low-m5Cscore groups in I (202 samples), II (680 samples), III (575 samples) and IV (135 samples).

Although our above results demonstrated the vital function of m5C methylation modification in immune infiltration modulation and prognosis, these analyses were only based on the CRC patient population and could not accurately predict the m5C modification patterns in individual patients. Therefore, a scoring scheme termed the “m5Cscore” was developed based on the identified m5C modification phenotype-related DEGs to quantify the m5C modification phenotype of individual CRC patients. Considering the complexity and heterogeneity of m5C modification, the m5Cscore was used to quantify m5C modification. All the m5Cscore values of the meta-CRC patients are summarized in Table S12, and an alluvial diagram was used to visualize the workflow of m5Cscore model construction and m5C modification phenotype changes in individual patients (Fig. 7A). We also investigated the m5Cscore difference in meta-CRC patients with distinct clinicopathological characteristics. The patients were divided into high and low m5Cscore groups with a cutoff value of 3.799 determined by the “survminer” package, and the high m5Cscore group showed a markedly better survival outcome than the low m5Cscore group (*P* < 0.0001, HR 0.63 (0.53–0.76)) (Fig. 5A). Patients with a high m5Cscore demonstrated a significant survival benefit in both age subgroups of patients (> 65 and ≤ 65 years of age) (Fig. 5F,G), and this survival benefit was also found in the stage subgroups, such as the stage II, stage III and stage VI subgroups (Fig. 5H–K). In addition, patients in stages III + IV showed lower m5Cscore values than patients in stages I + II (Fig. 5C), and the m5Cscore values in the nonsurviving patients were much lower than those in the surviving patients (Fig. 5D), but no significant differences were found among the distinct age groups (Fig. 5B). The diagram of the distribution of pathological stage among different m5Cscore groups shows that the high m5Cscore group includes more stage I + II patients, while the low m5Cscore group includes more stage III + IV patients (Fig. 5E). In summary, the m5Cscore may be identified as an independent prognostic biomarker for CRC patients.

### Analysis of the TME landscapes and biological processes of the distinct m5Cscore groups

**Figure 6:**
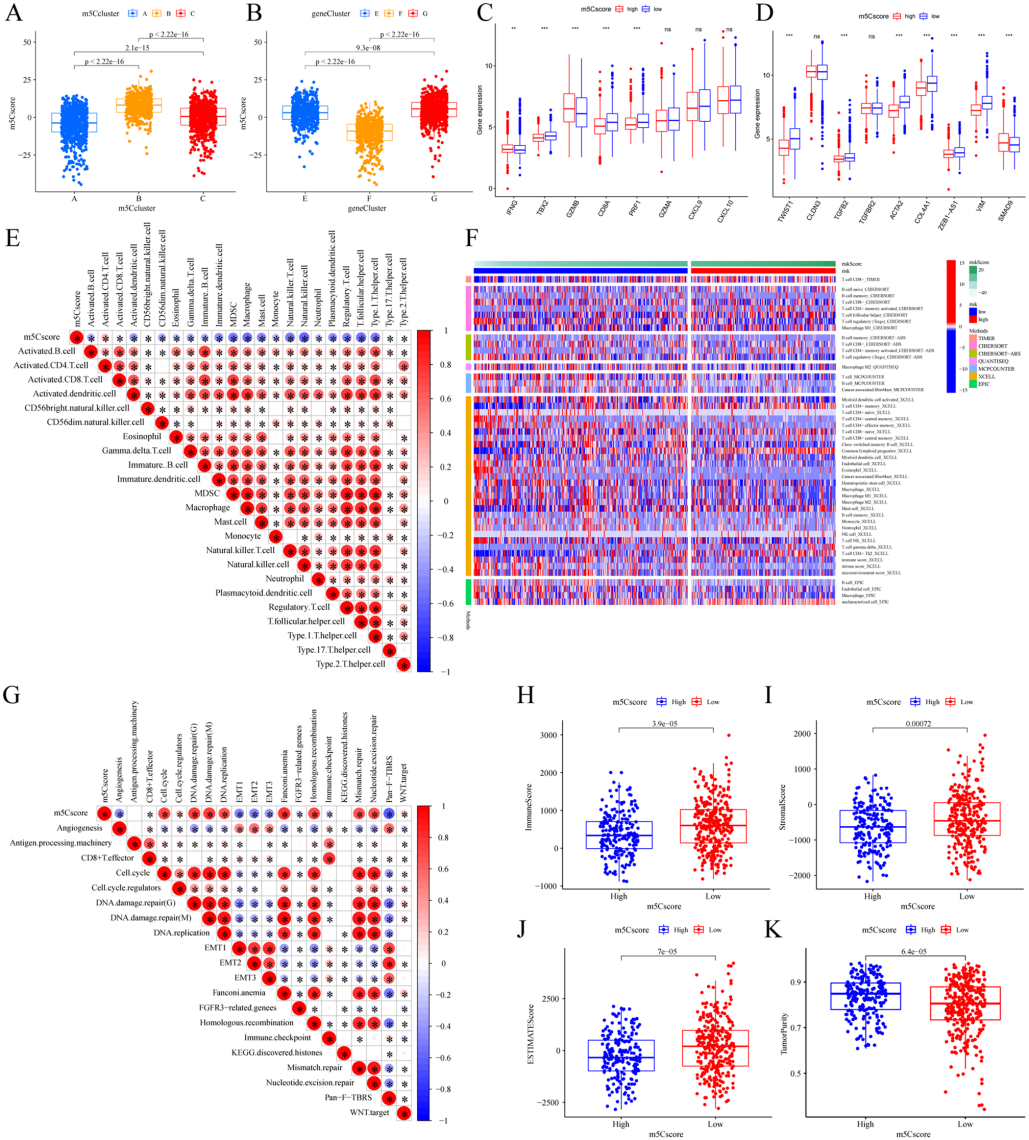
The TME landscapes and biological processes in the distinct m5Cscore groups. (**A**) The difference in the m5Cscore in the distinct m5C clusters using the Kruskal–Wallis test. (**B**) The difference in the m5Cscore in the distinct gene clusters. (**C**) The differential expression of each gene included in the immune activation-related gene signature between the m5Cscore high and low groups. (**D**) The differential expression of each gene included in the growth factor TGF-β/EMT pathway gene signature between the high and low m5Cscore groups. (**E**) The correlation between the m5Cscore and each TME-infiltrating cell type using Spearman analyses. The positive correlation is marked with red, and the negative correlation is marked with blue. The size of the circles represents the correlation coefficient (*P < 0.05). (**F**) Heatmap of the different immune cells that infiltrated the TME between the high and low m5Cscore groups. The clinical characteristics and CRC cohort distribution in the distinct m5Cscore groups. Each column represents patients, and each row represents individual immune cells. (**G**) Correlations between the m5Cscore and the known biological gene signatures using Spearman analyses. The positive correlation is marked with red, and the negative correlation is marked with blue. The size of the circles represents the correlation coefficient (*P < 0.05). (**H**) The different immune scores and three m5C clusters were compared and plotted. (**I**) Stromal score, (**J**) ESTIMATE score and (**K**) tumor purity between the high and low m5Cscore groups.

We further investigated the association of the m5Cscore with the TME landscape and biological processes. First, the Kruskal–Wallis test was used to reveal the difference among the three m5C clusters and the three m5C gene clusters. m5C cluster B had the highest median m5Cscore value, while m5C clusters A and C had lower median m5Cscore values (Fig. 6A). In contrast, gene cluster F showed a significantly lower m5Cscore than the other two gene clusters, and gene cluster G presented an increased median m5Cscore (Fig. 6B). More importantly, the chemokine and cytokine characteristics related to the m5Cscore were inspected according to enrichment of the TGF-β/EMT pathway and immune activation-related gene signature (Fig. 6C,D). As expected, the expression of most TGF-β/EMT pathway-related genes and immune activation-related genes was remarkably reduced in the m5Cscore high group compared with the low group, which further confirmed that a low m5Cscore could be closely linked to immune activation function. The relationship of the m5Cscore and each TME-infiltrating cell type using Spearman analyses and the obvious negative correlation between the m5Cscore and most TME-infiltrating cell types except CD4 + T cells in the ssGSEA result (Fig. 6E). The heatmap (Fig. 6F, Table S14) for immune cell infiltration showed that more immune cell infiltration was associated with the low m5Cscore group on different platforms. The ESTIMATE algorithm exhibited a higher immune score and Stormer score in the low m5Cscore group (Fig. 6H–K), which confirmed the above findings and demonstrated the crosstalk between the m5Cscore and immune infiltration evaluation. We examined the relationship between the m5Cscore and known biological processes through Spearman analysis. A heatmap of the correlation matrix (Fig. 6G) demonstrated that the m5Cscore score was highly positively correlated with the cell cycle, DNA damage repair, DNA replication, Fanconi anemia and mismatch repair but markedly negatively correlated with angiogenesis, EMT1-3 and Pan-F-TBRS signatures. All of these results strongly indicate that a low m5Cscore is significantly correlated with immune activation and indicates a better responses to ICI therapy. Compared to existing models, the m5Cscore could better for evaluating the m5C modification patterns of individual CRC patients and evaluating immune cell infiltration characteristics.

**Figure 7:**
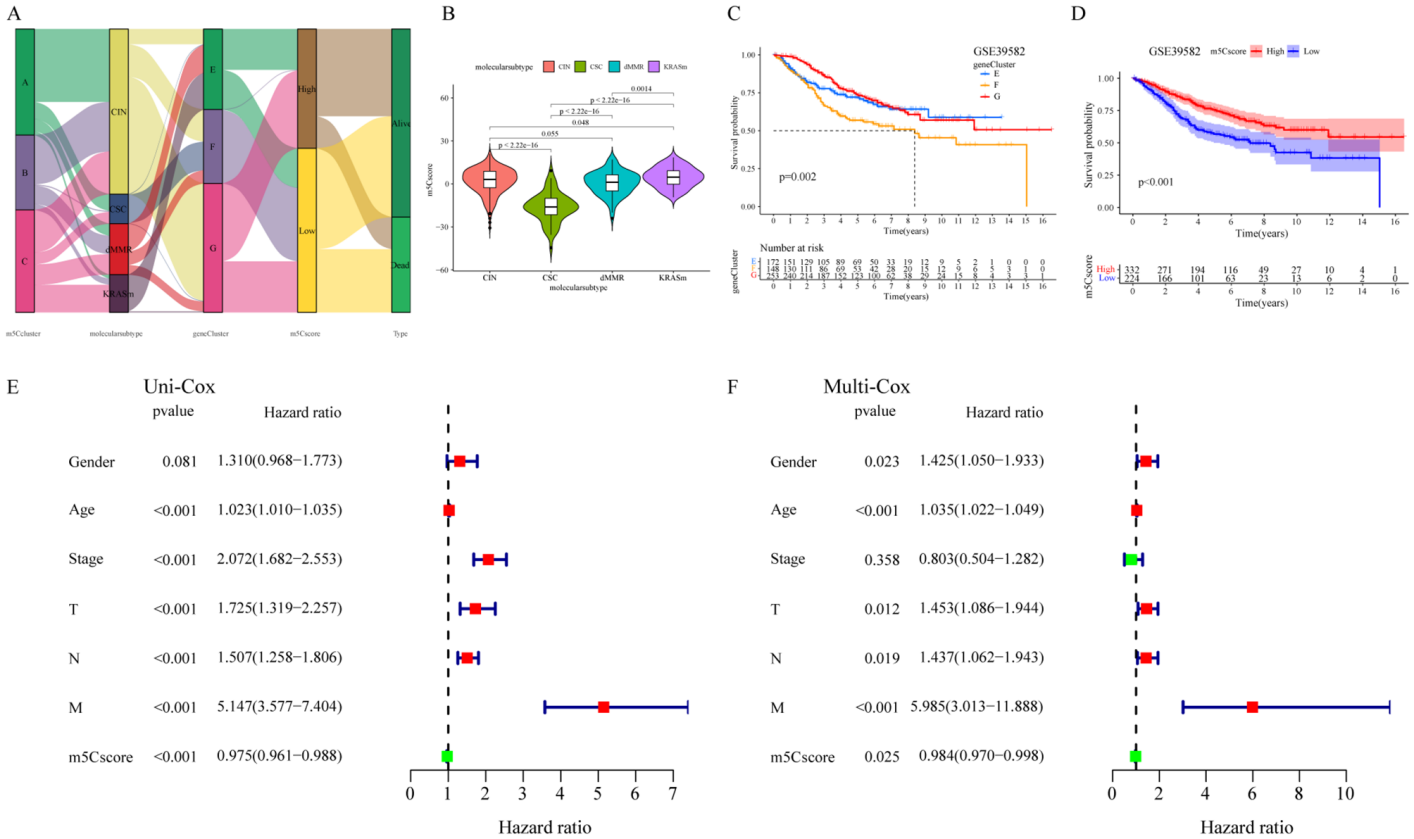
Characteristics of the m5C modification phenotype and m5Cscore and clinical value in the GSE39582 cohort. (**A**) Sankey diagram of the m5C clusters distributed to different molecular subtypes (dMMR, CSC, KRASm and CIN), m5C gene cluster, and m5Cscore. (**B**) The difference in the m5Cscore in the distinct molecular subtype groups (dMMR, CSC, KRASm and CIN). (**C**) The OS between the distinct m5C-related gene cluster groups. (**D**) The OS between the distinct high- and low-m5Cscore groups. (**E**) Univariate Cox regression analyses and (**F**) multivariate Cox regression analyses to estimate the ability of multiple clinical factors and the m5Cscore to predict OS. The length of the horizontal line represents the 95% confidence interval for each group. The vertical dotted line represents the HR of all patients. The vertical solid line represents HR = 1. The red square and hazard ratio > 1 represent risk factors for survival, and the green square and hazard ratio < 1 represent protective factors for survival.

Complete presentation matrix data, clinicopathological characteristics data, molecular subtypes data, and molecular marker data have been recorded for the GSE39582 cohort and were used to comprehensively validate the application value of the m5Cscore. The alluvial diagram indicated that the vast majority of gene cluster F and gene cluster G with the CIN subtype was linked to a lower m5Cscore, whereas gene cluster E and C with the dMMR or KRASm subtype exhibited a higher m5Cscore (Fig. 7A). The vast majority of CSC subtype samples were linked to gene cluster F and a lower m5Cscore. Furthermore, the m5Cscore in the KRASm subtype group was the highest, indicating the best survival, whereas the m5Cscore in the CSC subtype group was the lowest (Fig. 7B), indicating worse survival in these patients (Fig. S10E). The demonstrated survival benefit in patients with high m5Cscore values and the difference in survival trends in the distinct gene clusters were observed in the GSE39582 cohort and in the meta-CRC cohort (Fig. 7C,D). The m5Cscore showed no significant difference in the distinct molecular marker groups, such as the dMMR and pMMR, TP53 gene mutant and wild-type groups, and KRAS gene mutant and wild-type groups (Fig. S10A–C). In contrast, the m5Cscore in the patients who received adjuvant chemotherapy was markedly lower than that in the patients who did not receive adjuvant chemotherapy. Univariate Cox regression analysis and multivariate Cox regression analysis considering patient age, sex and TNM stage all confirmed the m5Cscore as a stable and independent prognostic biological biomarker (univariate Cox HR, 0.975 [95% CI, 0.961–0.975], *P* < 0.001, Fig. 7E; multivariate Cox HR, 0.984 [95% CI, 0.970–0.998], *P* = 0.025, Fig. 7F). Overall, the value of the m5Cscore was comprehensively demonstrated in CRC patients.

### Exploration of the role of the m5Cscore in predicting immunotherapeutic benefits

**Figure 8:**
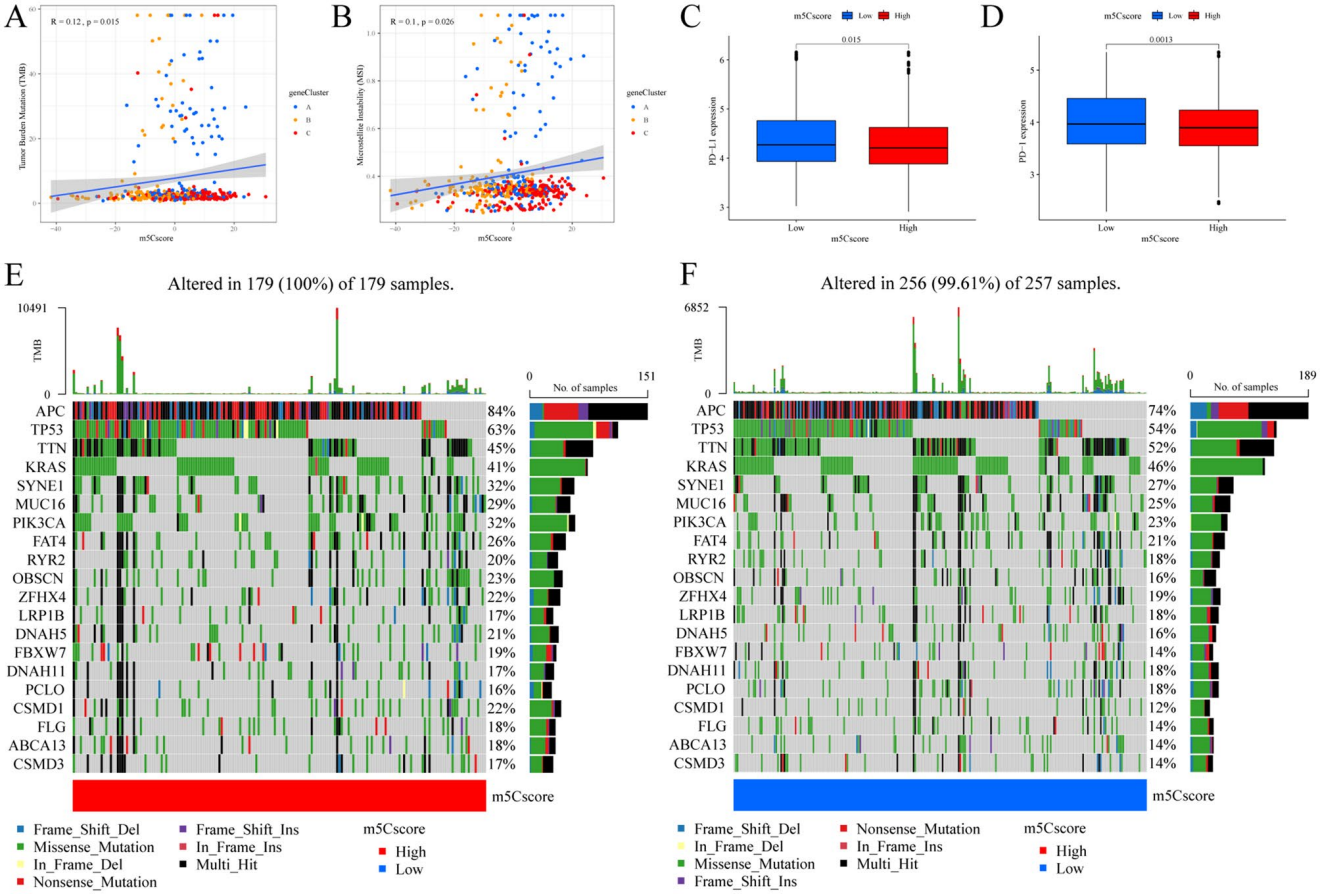
The role of the m5Cscore in predicting immunotherapeutic benefits. (**A**) The relationship between the m5Cscore and TMB. (**B**) The relationship between the m5Cscore and MSI. (**C**) PD-L1 expression and (**D**) PD-1 expression between the distinct high and low m5Cscore groups in the meta-CRC cohort. (**E**,**F**) Tumor somatic mutations were assessed in individuals in the high and low m5Cscore groups (shown in the left and right waterfall plots, respectively). The individual patients are represented in each column. The upper bar plot is TMB. The mutation frequency in each regulator is shown on the right. The proportion of each variant type is shown on the right bar plot. The fraction of conversions in each patient is shown in the stacked bar plot below.

Increasing evidence indicates that MSI and TMB can be used to predict immunotherapeutic responsiveness. However, the correlation coefficients of MSI (R = 0.1, *P* < 0.5) and TMB (R = 0.12, *P* < 0.5) with the m5Cscore were low (Fig. 8A,B). We further performed significantly mutated gene (SMG) analysis for CRC patients in the high m5Cscore subgroup versus the low m5Cscore subgroup. In addition, somatic mutations in the tumor genome were shown to be predictive markers of the immunotherapeutic response. The SMG mutational landscapes demonstrated that almost all of the SMGs had higher somatic mutation rates in the high m5Cscore subgroup, and the SMGs included APC (84 vs. 74%) and PIK3CA (32 vs. 23%) (*P* < 0.05, Fisher's exact test, Fig. 8E,F). In addition, compared with high TMB patients, low TMB patients had a better survival prognosis (*P* = 0.072, Fig. S10F). When we further stratified patients according to m5Cscore, patients with good survival prognosis could be better differentiated; for example, low TMB and high m5Cscore patients have the best prognosis, while patients with high TMB and low m5Cscore had the worst survival prognosis (*P* < 0.001, Fig. S10G). Combining TMB and the m5Cscore can improve patient prognosis prediction. We comprehensively revealed the effect of m5Cscore classification on genomic variation and the possible complex relationships between m5C modifications and individual tumor genome somatic mutations. Additionally, we found that the expression of each immune checkpoint gene [including PD-L1 (Fig. 8C) and PD-1 (Fig. 8D)] was markedly higher in the low m5Cscore group than in the high group (Fig. S11A).

### Verification of the predictive effect of the m5Cscore in terms of the ICI therapy response

**Figure 9:**
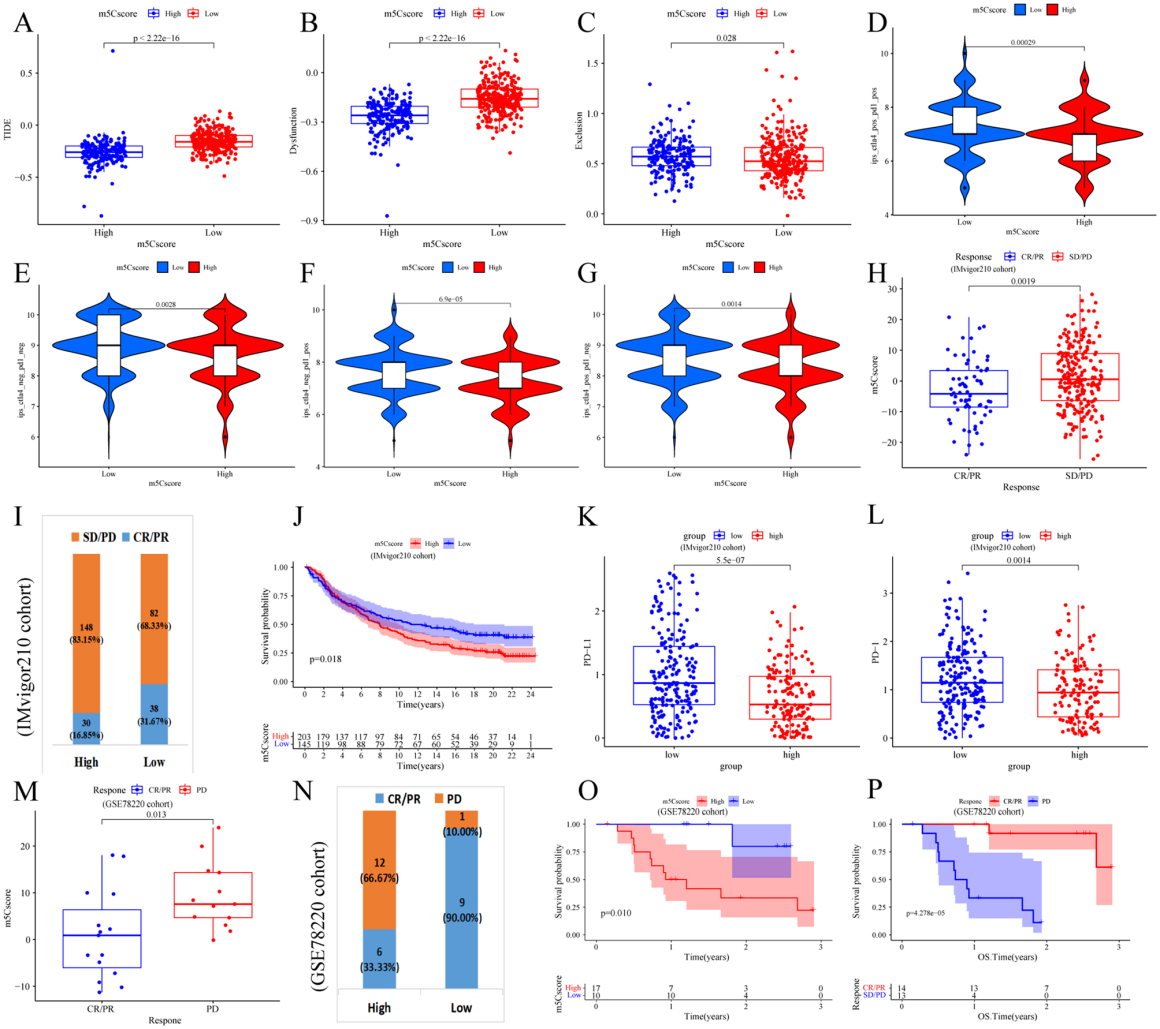
The predictive effect of the m5Cscore the ICI therapy response. (**A**–**C**) The difference in the TIDE scores between the high and low m5Cscore groups. (**D**–**G**) The difference in IPS between the high and low m5Cscore groups. (**H**–**L**) The prediction effect of anti-PD-L1 immunotherapy of m5Cscore in the IMvigor210 cohort. (**H**) The distribution of the m5Cscore between the patients with clinical response to anti-PD-L1 immunotherapy (CR, complete response; PR, partial response; SD, stable disease; PD, progressive disease). (**I**) The fraction of patients who responded to anti-PD-L1 immunotherapy in the high and low m5Cscore groups. (**J**) The survival analyses for low and high m5Cscore groups (K-M method, log-rank test). (**K**) PD-L1 expression and (**D**) PD-1 expression between the distinct high and low m5Cscore groups. (**M**–**P**) The prediction effect of the anti-PD-1 immunotherapy of m5Cscore in GSE78220 cohort. (**M**) The distribution of the m5Cscore between the patients with different clinical responses to anti-PD-1 immunotherapy. (**N**) The fraction of patients who responded to anti-PD-1 immunotherapy in the high and low m5Cscore groups. (**O**) The survival analyses for low and high m5Cscore groups (K-M method, log-rank test). (**P**) The survival analyses for the patients with distinct clinical response to anti-PD-1 immunotherapy groups (K-M method, log-rank test).

CTLA-4/PD-1 inhibitors are currently recognized as the most important and effective breakthrough ICI therapy method. The TIDE score and IPS are new and reliable predictors of the response to ICI therapy, and MSI, PD-L1, and TMB have been widely used as robust indicators; the correlations of these factors with the m5Cscore are shown. We further investigated the relationships between TIDE score, IPS and the m5Cscore. We also found that TIDE score was markedly elevated in the low m5Cscore group (both *P* < 0.001, Fig. 9A–C), and the IPS values were markedly higher in the low m5Cscore group than in the high group (both *P* < 0.001, Fig. 9D–G). All of the above findings indirectly demonstrate that tumor m5C modification patterns play a vital role in mediating immune infiltration and immune responses.

After we established the strong and reliable connection of the m5Cscore with the immune response, we further researched whether the m5C modification patterns and m5Cscore could be used to predict ICI therapy response in the IMvigor210 cohort treated with the anti-PD-L1 antibody and the GSE78220 cohort treated with the anti-PD-1 antibody. First, the m5Cscore values in the complete response (CR) and partial response (PR) to ICI therapy groups were markedly lower than those in the stable disease (SD) and progressive disease (PD) groups in both cohorts of patients (both *P* < 0.01, Fig. 9H,M). Next, the prominent therapeutic benefits and immunotherapeutic response to ICI treatment were confirmed in patients with low m5Cscore values compared to those with high m5Cscore values (CR and PR rates of the anti-PD-L1 cohort: 31.67 vs. 16.85%, Fig. 9I; CR and PR rates of the anti-PD-1 cohort: 90.0 vs. 33.33%, Fig. 9N). Finally, remarkable clinical advantages and conspicuous prolonged survival were found and exhibited in patients with low m5Cscore values (anti-PD-1, HR, 1.38 [95% CI, 0.106–1.81], *P* = 0.018). Figure 9J; anti-PD-1, HR, 9.37 [95% CI, 1.19 to 73.67], *P* = 0.01, Fig. 9O). Thus, this survival benefit was closely related to the survival benefit of responding to immunotherapy (Fig. 9P). In addition, the expression of PD-L1 and PD-1 in the low m5Cscore patients was obviously higher than that in the high m5Cscore patients in the IMvigor210 cohort, so it was also demonstrated to be a potential anti-PD-1 or anti-PD-L1 immunotherapy response biomarker (Fig. 9K,L); however, this difference was not found in the GSE78220 cohort, perhaps due to its small sample size (Fig. S11B). We also assessed the immune phenotypes of patients in the IMvigor210 cohort and further revealed the difference in the m5Cscore among distinct immune phenotype groups. The m5Cscore was the lowest in the inflamed immune phenotype patients and the highest in the exclusion immune phenotype patients (Fig. S11C), which further demonstrates the important role of the m5Cscore in identifying m5C modification patterns and other molecular modification patterns of individuals. In summary, our findings strongly indicate that the m5C methylation modification patterns and the established m5Cscore are closely associated with the response to anti-PD-1/L1 immunotherapies and can further predict the response to anti-PD-1/L1 immunotherapy in individual CRC patients.

## Discussion

With the continuous advancement of m5C detection methods and technologies, there is an increasing number of functional studies of m5C regulators^4^. Increasing evidence shows that multiple mRNA m5C regulators and m5C modifications play indispensable roles in the development and progression of tumors, specifically in cancer cell proliferation and metastasis, tumor TME shaping and cancer stem cell development, by regulating mRNA expression, translation and stability^8–10^. However, most of the current research is limited to one or two m5C regulators, but the construction and remodeling of the tumor TME is regulated by multiple regulators and multiple molecules in a highly coordinated manner. Therefore, a comprehensive understanding of the TME cell infiltration properties and TME landscape that are coordinated and mediated by multiple m5C regulators by identifying distinct m5C modification phenotypes will help to enhance our understanding of tumor immunotherapy.

Here, three distinct m5C modification phenotypes were identified based on 14 m5C regulators, and the different infiltrated immune cells in the TME were characterized and distinguished. The m5C cluster C was clearly recognized as an immune-inflamed phenotype significantly related to abundant infiltrating immune cells and immune activation, m5C cluster B was recognized as an immune-desert phenotype significantly related to infiltrating a lack of immune cells, and m5C cluster A was recognized as an immune-excluded phenotype significantly related to stromal cell, EMT and TGF-β pathway activation and suppressed immune cell infiltration. The immune-desert and immune-excluded phenotypes refer to noninflamed “cold” tumors, while the immune-inflamed phenotype refers to “hot” tumors^39,40^. The immune-excluded phenotype has a better chance of being transformed into a hot tumor based on the presence of many immune cells. Rather than penetrating the stroma and infiltrating the tumor parenchyma, these immune cells often remain only in the stroma surrounding tumor cell nests. Many structures and components of the stroma may be confined to the tumor capsule or may penetrate and segment the tumor tissue itself, making these cells appear to surround the tumor^41,42^. Many studies have shown that the tumor microenvironmental background plays a crucial role in tumor progression, patient survival prognosis, and the efficacy of immunotherapy^20^. The levels of infiltrating CD4 + /CD8 + T cells, Tregs, M1 macrophages, and NK cells in tumor tissues have been shown to be closely related to antitumor immune responses^43–45^. There are many antitumor effector immune cells, such as CD8 + T cells, NK cells, Th17 cells, Th2 cells and Tregs, that infiltrate the TME of m5C cluster C samples, which may be the reason this group has the best prognosis. The m5C cluster C phenotype was remarkably associated with increased levels of PD-L1 expression and TME-infiltrating lymphocytes and has potential predictive value in predicting a favorable response to ICI immunotherapy. Recent studies revealed that activation of TGF-β and EMT-related pathways may impede lymphocyte penetration into the tumor parenchyma^46^. We also found that m5C cluster A patterns were associated with activation of the TGF-β and EMT-related pathways. As a negative regulator of antitumor immunity, TGF-β impairs anti-PD-1/PD-L1 efficacy and induces drug resistance^47^. Yi Ming^48^ developed a drug YM101 that blocks the TGF-β signaling pathway, which can convert cold tumors into hot tumors, increase the number of tumor-infiltrating lymphocytes and enhance antitumor immune responses in the TME, contributing to better antitumor effects. The significance of blocking the TGF-β signaling pathway in remodeling the immune microenvironment and enhancing sensitivity to anti-PD-1/PD-L1 therapy can be found. According to these findings, we speculate that CRC patients with an m5C cluster A pattern may benefit from combined therapy with TGF-β blockade and anti-PD-1/PD-L1 drugs.

Next, we further investigated DEGs related to different m5C modification phenotypes, most of which were significantly enriched for biological pathways involved in mRNA modification and immunity, suggesting that these DEGs are considered to be an m5C phenotype-related feature gene signature. Using m5C phenotype-associated DEGs, we identified three gene cluster subtypes that were similar to the m5C modification clusters and were significantly associated with different patient survival outcomes and TME landscapes. The immune cell distribution among the gene cluster subtypes was more consistent and more significantly associated with stromal and immune activation and known biological property datasets than the m5C cluster phenotypes. This again proves that the m5C modification mode plays an important role in the development of the TME. Therefore, a comprehensive assessment of m5C modification phenotypes will provide insight and an understanding of TME cell infiltration characteristics and their role in immunotherapy and immune function assessment. We further constructed a quantitative model named the "m5Cscore" by the PCA method to define different m5C modification patterns in order to quantify m5C modification phenotypes more accurately and guide treatment strategies for individual patients. The m5Cscore was negatively correlated with the vast majority of TME-infiltrating immune cells. The m5C modification pattern characterized by the immune desert phenotype exhibited the highest m5Cscore, while the immune excluded phenotype m5C modification pattern exhibited the lowest m5Cscore. Further research revealed that the m5Cscore may be a favorable prognostic marker for CRC. It is closely related to the mutation signature and MSI score, which are often considered important indicators of immunotherapy response^49,50^, indicating that the m5Cscore may also be a surrogate indicator for evaluating the immunotherapy response. We also observed that the m5Cscore was closely related to other predictors of the immune response, including immune checkpoint genes such as PD-L1 and IPS and TIDE score, implying that m5C modification may affect the efficacy of immunotherapy treatment. In fact, we determined the strong predictive power of the m5Cscore in the immune response using two independent cohorts of ICI-treated patients. Interestingly, in both independent cohorts of ICI-treated patients, the lower m5Cscore group was found to have a better response to ICI treatment, as well as a better prognosis. In contrast, patients with high m5Cscore values had a better prognosis in the large meta-CRC cohort. This difference in survival prognosis may be due to treatment with ICIs, and this treatment response is more durable and brings a more pronounced favorable survival benefit over traditional treatments, supporting our hypothesis that m5C modification patterns can be used in clinical practice to classify patients according to immunophenotypes and guide treatment regimens.

In addition to elucidating the role of m5C modification clustering, we discussed the internal mechanism and role of a single m5C regulator in regulating tumor immunity. TET2, a member of the ten-eleven translocation (TET) family, mediates the oxidation of mRNA m5C as α-ketoglutaric acid, and Fe-dependent dioxygenase catalyzes the iterative oxidation of m5C^51^. TET2 is considered an eraser of m5C^52^ and plays an important role in maintaining epigenome plasticity and in cancer development^53^. Mutations and disorders of TET2 lead to the development of various malignancies and may be important regulators of immune cell function^54^. TET2, which is considered a tumor suppressor gene in a variety of tumors^55^, may play a role in inhibiting tumor growth and development by adjusting the infiltration and function of immune cells in the tumor immune microenvironment through many mechanisms^56,57^. Kang Dong Hyun's study^58^ found that patients with low TET2 expression levels more frequently developed distant metastases, but there was no significant correlation with other clinicopathological characteristics of the patients (age, tumor location, vascular, lymphatic and perineural invasion and TNM stage). A high expression level of TET2 indicated a better survival prognosis than a low TET2 level and was an independent predictor of good prognosis. This was also consistent with the results in our study; TET2 was significantly downregulated in CRC tissues and was a protective factor for patient survival prognosis. Further analysis showed that the expression of TET2 was positively correlated with the level of immune cell infiltration and the expression level of immune activation-related genes, suggesting that TET2 regulates immune cell infiltration and activation of immune cells in CRC. Moreover, the high TET2 expression group had a higher score of CD8 + T-cell effector activation in our research, and the possible underlying mechanism is that TET2 may mediate the IL-6/G-MDSC/CD8 + T-cell immune cascade and protect adaptive host antitumor immunity^22^. TET2 can mediate the interferon gamma (IFNγ)-JAK-STAT signaling pathway to control chemokine and PD-L1 expression, lymphocyte infiltration, and cancer immunity^59^. Reduced TET2 activity is associated with reduced TH1-type chemokines and tumor-infiltrating lymphocytes (TILs) and the progression of human colon cancer. Loss of TET2 in colon tumor cells reduced chemokine expression and the number of TILs, enabling tumors to evade antitumor immunity and resist anti-PD-L1 therapy. TET2 activity can be used as a biomarker to predict efficacy and the response to anti-PD-1/PD-L1 therapy, and stimulation and enhancement of TET2 activity can be used as adjuvant immunotherapy strategy for solid tumors. More results demonstrated the crucial role of TET2 in remodeling the TME and improving the immunotherapy response^23^. We also revealed that the majority of m5C writers were negatively correlated with the number of infiltrating immune cells. In contrast, the expression of the m5C-reader ALYREF was significantly positively correlated with the infiltration of lymphocytes such as CD4 + T cells, CD8 + T cells, DCs and NK cells, which may be the basis for the role of m5C regulators in tumorigenesis and development. Together, these results demonstrate the heterogeneity of m5C modification and its role in regulating immune cell infiltration in the tumor microenvironment, demonstrate the importance of comprehensively assessing m5C modification patterns, and enhance our understanding of m5C modification and other related epigenetic regulatory mechanisms. This provides an understanding of the roles played by different physiological processes.

Recently, an increasing number of m5C regulators have been confirmed and studied. We need to further incorporate more newly discovered m5C regulators into this model to optimize the accuracy of m5C modification patterns and improve the application value of this model. At present, there is no relevant dataset suitable for the application of ICIs in CRC. We can only use the immunotherapy datasets of other tumors (melanoma and urothelial carcinoma) to verify the effect of the m5Cscore in predicting the treatment response of ICIs to further strengthen our conclusion. We are looking forward to a prospective randomized controlled cohort study of CRC patients receiving immunotherapy to evaluate the role of the m5Cscore. In addition, we can combine the m5Cscore with other molecular indicators, clinicopathological characteristics and other indicators, and by comparing multiple gene set system scoring methods including PCA, sigQC^60,61^, etc., the optimal method can be selected for the construction of prediction model to build a predictive model to improve the accuracy of model prediction and its clinical application value.

## Conclusion

In conclusion, the m5Cscore can be used in clinical practice to comprehensively evaluate the m5C methylation modification phenotypes of individual CRC patients and their corresponding TME cell infiltration characteristics and to classify them based on the immunophenotype of CRC to guide in the selection of more appropriate treatment strategies, which has important clinical application value. The specific relationships between the m5Cscore and the clinicopathological characteristics of patients (such as clinical stage, genetic variation, molecular subtype, MSI levels, TMB levels, immune checkpoint gene expression, etc.) and the characteristics of TME immune infiltrating cell roles were fully demonstrated in our study. The m5Cscore can also predict the clinical response of patients to anti-PD-1/PD-L1 immunotherapy, and patients with a low m5Cscore, especially those with a low m5Cscore and the immune excluded phenotype, may respond to ICI treatment to improve their survival. This also brings some new insights into cancer immunotherapy research, targeting m5C regulators or m5C phenotype-related genes to alter m5C modification patterns and further reverse the patient's unfavorable TME cell infiltration signature in the immune excluded subtype. The transformation of cold tumors into hot tumors improves the efficacy immunotherapy in the immune excluded group and may contribute to the development of new drugs related to m5C modification patterns in the future or an effective anticancer strategy for new immunotherapeutic combinations. Our findings can be used not only to identify different tumor immunophenotypes and evaluate patients' clinical response to immunotherapy but also to improve immunotherapy for patients with immune excluded subtypes of cancer and promote the application of personalized immunotherapy in the future.

## Supplementary Information

Supplementary Figures.

Supplementary Tables.

## Data Availability

The datasets generated and analyzed during the current study are available in the TCGA repository [https://portal.gdc.cancer.gov/] and the GEO repository [www.ncbi.nlm.nih.gov/geo/]. We declare that the data and materials in this study are provided free of charge to scientists for noncommercial purposes.

## References

[1] Pietro B, Filip S, Angana R (2021). MODOMICS: A database of RNA modification pathways. Nucleic Acids Res..

[2] Phensinee H, Ying ZY, Yubin Z (2020). RNA modifications and cancer. RNA Biol..

[3] Rong H, Changfeng M, Jiabin H (2020). Identification of RNA methylation-related lncRNAs signature for predicting hot and cold tumors and prognosis in colon cancer. Front Genet..

[4] Gangqiang G, Kan P, Fang Su (2021). Advances in mRNA 5-methylcytosine modifications: Detection, effectors, biological functions, and clinical relevance. Mol. Ther. Nucleic Acids.

[5] Tuorto F, Liebers R, Musch T (2012). RNA cytosine methylation by Dnmt2 and NSun2 promotes tRNA stability and protein synthesis. Nat. Struct. Mol. Biol..

[6] Chan CT, Pang YL, Deng W (2012). Reprogramming of tRNA modifications controls the oxidative stress response by codon-biased translation of proteins. Nat. Commun..

[7] Shanmugam R, Fierer J, Kaiser S (2015). Cytosine methylation of tRNA-Asp by DNMT2 has a role in translation of proteins containing poly-Asp sequences. Cell Discov..

[8] Zhenhuan Lv, Chunli X, Lei Z (2021). Elevated mRNA level of Y-box binding protein 1 indicates unfavorable prognosis correlated with macrophage infiltration and T cell exhaustion in luminal breast cancer. Cancer Manag. Res..

[9] Hailong R, Lin B, Zhen T (2021). Flightless I homolog reverses enzalutamide resistance through PD-L1-mediated immune evasion in prostate cancer. Cancer Immunol. Res..

[10] Chen Q, Zuyin L, Wanyue C (2021). Correlation analysis of RDM1 gene with immune infiltration and clinical prognosis of hepatocellular carcinoma. Biosci. Rep..

[11] Feifei Li, Yang Z, Youyang S (2021). Comprehensive analysis of prognostic and immune infiltrates for RAD51 in human breast cancer. Crit. Rev. Eukaryot. Gene Expr..

[12] Siegel Rebecca L, Miller Kimberly D, Fuchs Hannah E (2021). Cancer statistics, 2021. CA Cancer J. Clin..

[13] Hyuna S, Jacques F, Rebecca LS (2020). Global cancer statistics 2020: GLOBOCAN estimates of incidence and mortality worldwide for 36 cancers in 185 countries. CA Cancer J. Clin..

[14] Parul A, Le Dung T, Boland Patrick M (2021). Immunotherapy in colorectal cancer. Adv. Cancer Res..

[15] Mei F, Zhongwei Z, Mengxuan Y (2021). T-cell-based immunotherapy in colorectal cancer. Cancer Lett..

[16] Toor SM, Nair VS, Decock J, Elkord E (2020). Immune checkpoints in the tumor microenvironment. Seminars in cancer biology.

[17] Camus M, Tosolini M, Mlecnik B, Pages F, Kirilovsky A, Berger A (2009). Coordination of intratumoral immune reaction and human colorectal cancer recurrence. Cancer Res..

[18] Topalian Suzanne L, Taube Janis M, Anders Robert A (2016). Mechanism-driven biomarkers to guide immune checkpoint blockade in cancer therapy. Nat. Rev. Cancer.

[19] Chen DS, Mellman I (2017). Elements of cancer immunity and the cancer-immune set point. Nature.

[20] Galon J, Bruni D (2019). Approaches to treat immune hot, altered and cold tumours with combination immunotherapies. Nat. Rev. Drug Discov..

[21] Li X, Wen D, Li X, Yao C (2020). Identification of an immune signature predicting prognosis risk and lymphocyte infiltration in colon cancer. Front Immunol..

[22] Shuangqi Li, Jiuxing F, Wu F (2020). TET2 promotes anti-tumor immunity by governing G-MDSCs and CD8 T-cell numbers. EMBO Rep..

[23] Ding P, Anbang He, Shiming He (2022). Ascorbic acid induced TET2 enzyme activation enhances cancer immunotherapy efficacy in renal cell carcinoma. Int. J. Biol. Sci..

[24] Jin Z, Weiqing J, Orentas Rimas J (2009). Discovery of YB-1 as a new immunological target in neuroblastoma by vaccination in the context of regulatory T cell blockade. Acta Biochim. Biophys. Sin. (Shanghai).

[25] Xiaoxuan C, Miaomiao L, Di Li (2021). RAN and YBX1 are required for cell proliferation and IL-4 expression and linked to poor prognosis in oral squamous cell carcinoma. Exp. Cell Res..

[26] Wilkerson MD, Hayes DN (2010). ConsensusClusterPlus: A class discovery tool with confidence assessments and item tracking. Bioinformatics.

[27] Hartigan JAWM (1979). Algorithm AS 136: A K-means clustering algorithm. Appl. Stat..

[28] Ritchie ME, Phipson B, Wu D (2015). Limma powers differential expression analyses for RNA-sequencing and microarray studies. Nucleic Acids Res..

[29] Zhang Bo, Qiong Wu, Li B (2020). m6A regulator-mediated methylation modification patterns and tumor microenvironment infiltration characterization in gastric cancer. Mol. Cancer.

[30] Wei C, Liang S, Jin L (2021). mA regulator-based methylation modification patterns characterized by distinct tumor microenvironment immune profiles in colon cancer. Theranostics.

[31] Zeng D, Li M, Zhou R (2019). Tumor microenvironment characterization in gastric cancer identifies prognostic and immunotherapeutically relevant gene signatures. Cancer Immunol. Res..

[32] Mariathasan S, Turley SJ, Nickles D (2018). TGFbeta attenuates tumour response to PD-L1 blockade by contributing to exclusion of T cells. Nature.

[33] Senbabaoglu Y, Gejman RS, Winer AG (2016). Tumor immune microenvironment characterization in clear cell renal cell carcinoma identifies prognostic and immunotherapeutically relevant messenger RNA signatures. Genome Biol..

[34] Rosenberg JE, Hoffman-Censits J, Powles T (2016). Atezolizumab in patients with locally advanced and metastatic urothelial carcinoma who have progressed following treatment with platinum-based chemotherapy: a single-arm, multicentre, phase 2 trial. Lancet.

[35] Barbie DA, Tamayo P, Boehm JS (2009). Systematic RNA interference reveals that oncogenic KRAS-driven cancers require TBK1. Nature.

[36] Charoentong P, Finotello F, Angelova M (2017). Pan-cancer immunogenomic analyses reveal genotype-immunophenotype relationships and predictors of response to checkpoint blockade. Cell Rep..

[37] Jiang P, Gu S, Pan D (2018). Signatures of T cell dysfunction and exclusion predict cancer immunotherapy response. Nat. Med..

[38] Hugo W, Zaretsky JM, Sun L (2016). Genomic and transcriptomic features of response to anti-PD-1 therapy in metastatic melanoma. Cell.

[39] Marisa L, de Reynies A, Duval A (2013). Gene expression classification of colon cancer into molecular subtypes: Characterization, validation, and prognostic value. PLoS Med..

[40] Turley SJ, Cremasco V, Astarita JL (2015). Immunological hallmarks of stromal cells in the tumour microenvironment. Nat. Rev. Immunol..

[41] Gajewski TF (2015). The next hurdle in cancer immunotherapy: Overcoming the non-T-cell-inflamed tumor microenvironment. Semin. Oncol..

[42] Joyce JA, Fearon DT (2015). T cell exclusion, immune privilege, and the tumor microenvironment. Science.

[43] Alexandros L, Ali T, Nadia T (2021). Prognostic significance of CD8+ T-cells density in stage III colorectal cancer depends on SDF-1 expression. Sci. Rep..

[44] Hang LJ, Michelle H, Si-Lin K (2021). CD30OX40 Treg is associated with improved overall survival in colorectal cancer. Cancer Immunol Immunother.

[45] Zeng D, Ye Z, Wu J, Zhou R, Fan X, Wang G (2020). Macrophage correlates with immunophenotype and predicts anti-PD-L1 response of urothelial cancer. Theranostics.

[46] Tauriello DVF, Palomo-Ponce S, Stork D, Berenguer-Llergo A, Badia-Ramentol J, Iglesias M (2018). TGFbeta drives immune evasion in genetically reconstituted colon cancer metastasis. Nature.

[47] Zhang Xue-Li Hu, Li-Peng YQ (2021). CTHRC1 promotes liver metastasis by reshaping infiltrated macrophages through physical interactions with TGF-β receptors in colorectal cancer. Oncogene.

[48] Ming Yi, Jing Z, Anping Li (2021). The construction, expression, and enhanced anti-tumor activity of YM101: A bispecific antibody simultaneously targeting TGF-β and PD-L1. J. Hematol. Oncol..

[49] Peter S (2021). MSI-H/dMMR mCRC: ICIs in the first line?. Nat. Rev. Clin. Oncol..

[50] Xuan Z, Tao W, Xinyi C (2022). Neoadjuvant immunotherapy for MSI-H/dMMR locally advanced colorectal cancer: New strategies and unveiled opportunities. Front Immunol..

[51] Shen Q, Zhang Q, Shi Y (2018). Tet2 promotes pathogen infection-induced myelopoiesis through mRNA oxidation. Nature.

[52] Jie L, Nicholas R, Martin B (2020). Functional role of Tet-mediated RNA hydroxymethylcytosine in mouse ES cells and during differentiation. Nat. Commun..

[53] Elise B, Enrico R, Martin B (2019). TET2-dependent hydroxymethylome plasticity reduces melanoma initiation and progression. Cancer Res..

[54] Boyi C, Qian Z, Xuetao C (2021). The function and regulation of TET2 in innate immunity and inflammation. Protein Cell.

[55] Yang SX, Hollingshead M, Rubinstein L (2021). TET2 and DNMT3A mutations and exceptional response to 4'-thio-2'-deoxycytidine in human solid tumor models. J. Hematol. Oncol..

[56] Chengshun Li, Chuanni P, Ziping J (2022). Ginkgo biloba extract inhibited cell proliferation and invasion by stimulating TET2 expression through miR-29a in colorectal carcinoma cells. DNA Cell Biol..

[57] Hulin Ma, Weishi G, Xiaoxia S (2018). STAT5 and TET2 cooperate to regulate FOXP3-TSDR demethylation in CD4 T cells of patients with colorectal cancer. J. Immunol. Res..

[58] Hyun KD, Jun JD, Sung AT (2021). Expression of AMP-activated protein kinase/ten-eleven translocation 2 and their clinical relevance in colorectal cancer. Oncol. Lett..

[59] Yan-Ping Xu, Lei Lv, Ying L (2019). Tumor suppressor TET2 promotes cancer immunity and immunotherapy efficacy. J. Clin. Invest..

[60] Dhawan A, Scott J, Sundaresan P, Veness M, Porceddu S, Hau E, Harris AL, Buffa FM, Gee HE (2020). Role of gene signatures combined with pathology in classification of oropharynx head and neck cancer. Sci. Rep..

[61] Dhawan A, Barberis A, Cheng WC, Domingo E, West C, Maughan T, Scott JG, Harris AL, Buffa FM (2019). Guidelines for using sigQC for systematic evaluation of gene signatures. Nat. Protoc..

