# Supplementary material for: m5C regulator-mediated methylation modification phenotypes characterized by distinct tumor microenvironment immune heterogenicity in colorectal cancer
Source: Sci Rep. 2023 Jul 24;13:11950. doi: 10.1038/s41598-023-37300-z (PMC10366215; doi:10.1038/s41598-023-37300-z)
Supplement: Supplementary file 1 — Supplementary Figures. [file 41598_2023_37300_MOESM1_ESM.doc]

**Figure S1. Overview of this entire analysis workflow.** The biorender app (online) was used to create this figure. (https://app.biorender.com/)

**Figure S2.** **Mutation cooccurrence and expression differences between mutant and wild-type groups of 14 m5C regulators.** **(A)** The mutation cooccurrence and exclusion analyses for 14 m5C regulators in TCGA-CRC. Co-occurrence, green; exclusion, brown. **(B-E)** The significantly differentially expressed m5C regulators (ALYREF, DNMT3A, NSUN4 and NSUN6) in the mutant and wild-type groups (p< 0.05). **(F-O)** The other nonsignificantly differentially expressed m5C regulators in the mutant and wild-type groups (p> 0.05).

**Figure S3.** **The prognostic analysis of 14 m5C regulators and their expression in the m5C clusters.** **(A)** Univariate Cox regression results estimating the clinical prognostic significance of the 14 m5C regulators. The length of the horizontal line represents the 95% confidence interval for each group. The vertical dotted line represents the HR of all patients. The vertical solid line represents HR = 1. The red square and hazard ratio >1 represent risk factors for survival, and the blue square and hazard ratio < 1 represent protective factors for survival. **(B-L)** Survival analyses for patients with low or high m5C regulator expression in 1609 meta-CRCs with significantly different survival results of the K-M method (p<0.05). **(M)** The expression of the 14 m5C regulators in the distinct m5C clusters. (*P < 0.05; **P < 0.01; ***P < 0.001.).

**Figure S4.** **Unsupervised clustering was performed for 14 m5C regulators and the differential expression of biological characteristic-related genes among the m5C clusters.** **(A-E)** Consensus matrices of the 1609 meta-CRC cohort for k = 2-6. **(F)** The consensus CDF with respect to the number of clusters. **(G)** The delta area of consensus CDF with respect to the number of clusters. **(H)** The tracking plot of clusters. **(I)** Differences in immune activation-related gene expression among the three m5C clusters. **(J)** The difference in transforming growth factor TGF-β/EMT pathway-related gene expression among the three m5C clusters.

**Figure S5.** **The result of KEGG in GSVA between different m5C clusters.** **(A)** Heatmap of the different functional annotations of KEGG between m5C cluster A and m5C cluster B. (**B)** Heatmap of the different functional annotations between m5C cluster A and m5C cluster C. **(C)** Heatmap of the different functional annotations between m5C cluster B and m5C cluster C. **(D)** The Kruskal–Wallis H test was used to statistically analyze and plot the difference between distinct clusters. The checkpoint gene **(E)** PD-1, **(F)** CTLA4 and **(G)** CD80 expression in the distinct m5C clusters. **(H)** Differences in immune checkpoint-related gene expression among the three m5C clusters.

**Figure S6.** **Unsupervised clustering was performed for m5C regulator-related DEGs (m5C gene cluster) and the differential expression of biological characteristic-related genes among the gene clusters.** **(A-E)** Consensus matrices of the 1609 meta-CRC cohort patients for k = 2-6. **(F)** The consensus CDF with respect to the number of clusters. **(G)** The delta area of consensus CDF with respect to the number of clusters. **(H)** The tracking plot of clusters. **(I)** Differences in immune activation-related gene expression among the three gene clusters. **(J)** The difference in the transforming growth factor TGF-β/EMT pathway-related gene expression among the three gene clusters.

**Figure S7. The distinct TME characteristics between the three gene cluster phenotypes.** Heatmap of the different of the known biological processes among **(A)** the three m5C clusters and **(B)** the three gene clusters. The clinical characteristics and CRC cohort distribution in the distinct groups. Each column represents patients, and each row represents individual known biological gene signatures. **(C)** Heatmap of the different immune cells that infiltrated the TME between the three gene cluster phenotypes. The clinical characteristics and CRC cohort were distributed in distinct m5C clusters. Each column represents patients, and each row represents individual immune cells. The Kruskal–Wallis H test was used to statistically analyze and plot the differences between distinct clusters. **(D)** The immune score, **(E)** stromal score, **(F)** ESTIMATE score and **(G)** tumor purity of the three gene clusters. The checkpoint gene **(H)** PD-L1, **(I)** PD-1, **(J)** CTLA-4 and **(K)** CD80 expression in the distinct gene clusters. **(L)** Differences in immune checkpoint-related gene expression among the three gene clusters.

**Figure S8. The relationship of m5C regulators and infiltrated immune cells and known biological processes.** **(A)** Heatmap of the correlation between each different immune cell infiltrate in the TME and each m5C regulator using Spearman analyses. **(B)** Heatmap of the correlation between the enrichment score of each known biological process gene signature and each m5C regulator using Spearman analyses. The negative correlation is shown in blue, and the positive correlation is shown in red (*P < 0.05 and **P < 0.01).

**Figure S9. The distinct characteristics of the m5C modification eraser TET2 in the meta-CRC cohort.** **(A)** The difference in each different immune cell infiltrate in the CRC TME between the median cut of the TET2 high and low expression groups. **(B)** The difference in the enrichment score of each known biological process gene signature between the TET2 high and low expression groups. The difference in **(C)** the immune score, **(D)** stromal score, **(E)** ESTIMATE score and **(F)** tumor purity score between the TET2 high and low expression groups. **(G)** The differential expression of each m5C regulator between the TET2 high and low expression groups. **(H)** The differential expression of each gene included in the immune activation-related gene signature between the TET2 high and low expression groups. **(I)** The differential expression of each gene included in the growth factor TGF-β/EMT pathway gene signature between the TET2 high and low expression groups. **(J)** The differential expression of each immune checkpoint gene between the TET2 high and low expression groups. (*P < 0.05, **P < 0.01 and ***P < 0.001).

**Figure S10. The relationship between the m5Cscore and common molecular mutations and the analysis of survival based on TMB in the GSE39582 cohort.** **(A)** The difference in the m5Cscore in dMMR and pMMR. **(B)** The difference in the m5Cscore in the KRAS gene mutant and wild-type groups. **(C)** The difference in the m5Cscore in the TP53 gene mutant and wild-type groups. **(D)** The difference in the m5Cscore in patients who received adjuvant chemotherapy. **(E)** Survival analysis of distinct molecular subtypes in the GSE39582 cohort. **(F)** Survival analysis of the high- and low-TMB groups. **(G)** The survival analysis of different TMB and m5Cscore groups.

**Figure S11.** **The difference of the immune checkpoint genes expression between distinct m5Cscore groups. (A)** The difference of the whole checkpoints genes expression in high and low m5Cscore groups in the meta-CRC cohort. **(B)** The difference of the PD-1 and CTLA-4 checkpoints expression in high and low m5Cscore groups in the GES78220 cohort. **(C)** The difference of m5Cscore in the immune phenotype groups in the IMvigor210 cohort.
